# Physiology and the future of animals in shifting-sand deserts: living, moving and thermoregulating in the sand

**DOI:** 10.1093/conphys/coag057

**Published:** 2026-08-20

**Authors:** Duncan Mitchell, Mary K Seely, Shane K Maloney

**Affiliations:** Department of Physiology, Brain Function Research Group, University of the Witwatersrand Medical School, 7 York Road, Parktown 2193, Johannesburg, South Africa; School of Human Sciences, University of Western Australia, 35 Stirling Highway, Crawley 6009, Perth, Australia; 55 Libertina Amathila Avenue, Swakopmund, Namibia; Department of Physiology, Brain Function Research Group, University of the Witwatersrand Medical School, 7 York Road, Parktown 2193, Johannesburg, South Africa; School of Human Sciences, University of Western Australia, 35 Stirling Highway, Crawley 6009, Perth, Australia

**Keywords:** Beetle, burrow, cold, desertification, heat, lizard, mole, sand-swimming

## Abstract

The conservation of the animals of the shifting-sand deserts requires a thorough understanding of the physiological mechanisms that not only enable them to function in the desert, but also may buffer them against future change. The animals there contend with little water and poor food resources. The animals of shifting-sand deserts also must move on and in loose sand and cannot construct unsupported burrows. Most are invertebrates, but the deserts also are home to many vertebrate species, mainly lizards. We describe the biomechanics of their locomotion on and in the sand. Some are quiescent buriers that remain near the surface, but remarkably, small desert mammals can swim to depths of more than 500 mm. Diurnally active animals retreat into the sand beneath their feet to avoid overheating in summer and freezing in winter, but burial and emergence are not determined by selection of the best thermal or hygric environment. We address the conundrum of why so many species that are active in the sun are black. The sand provides a fortress that resists invasion, but from which the residents cannot escape. The animals are under threat from climate change and from human use and abuse of the shifting-sand deserts, even though the sand is unsuitable for concrete. We argue that efforts to combat desertification do not absolve us from a need to conserve shifting-sand deserts. In a companion review, we discuss how the physiology of breathing, the acquisition of metabolic energy and the management of body water impacts the conservation of the animals of shifting-sand deserts.

This article is linked to “Physiology and the future of animals in shifting-sand deserts: respiration, energetics and water balance” (https://doi.org/10.1093/conphys/coag058).

## Introduction

The great shifting-sand deserts of the world include the Sahara, Taklamakan, Mojave, Sonoran, Arabian, Simpson, Thar, Negev, Karakum, Tengger and the Namib. What defines them as deserts is not their sand but their precipitation, typically a mean annual precipitation less than 100 mm per year (Noy-Meir, 1973) or potential evapotranspiration that exceeds the mean annual precipitation by at least 20 times (Le Houérou, 1996). Hyperarid deserts like the Namib Desert have had an annual mean rainfall of less than 20 mm for decades (Southgate *et al.*, 1996). While deserts are dry by definition, not all deserts are characterised by loose sand. They can be ice (Tedrow & Ugolini, 1966), predominantly firm soil like most of the Atacama Desert (Chile) and Junggar Basin (China), or covered by loose rocks and stones (‘gibber’), like the Sturt Stony Desert (Australia). We will be concerned here with a subset (perhaps 20%) of sandy deserts, namely those that have sand sufficiently loose and mobile that their surface-active animals always have a potential refuge in the substrate beneath their feet, but in which unsupported burrows cannot be constructed. We have called that subset of sandy deserts ‘shifting-sand deserts’; their loose mobile sand derives from aeolian forces, so they might also be called ‘aeolian deserts’. Though such deserts comprise a small proportion of the world’s deserts (Dawson *et al.*, 1989), 6–10% of the earth’s land surface is covered by sand (Maladen *et al.*, 2009). Those shifting-sand deserts look very similar in satellite images, but each desert has its own complex of flora and fauna (Seely, 1984). The age of the desert is an important (though not the only, Seely, 1979) determinant of its biodiversity, and the Namib Desert of southwestern Africa is the oldest (Ward *et al.*, 1983). Shifting-sand deserts provide special challenges to their animal inhabitants, and their adaptation to that habitat generates physiological lessons that can be learnt.

Animals survive in a habitat only when their physiology, including their behavioural responses, allows them to operate in that habitat. For conservation to materialise, physiology must succeed. The animals of the shifting-sand deserts have to cope physiologically with the challenges that face any desert animal, in particular, poor access to water and only the sparse vegetation that can exist in the aridity. In contrast to other habitats, the challenges to survival in a desert are more likely to be abiotic than biotic (Noy-Meir, 1973). There are thermal challenges, which can be challenges of heat and/or cold, and those animals that choose to use the shifting-sand refuge have to both swim and breathe in the sand.

In this review, and in a companion review (Mitchell *et al.*, 2026), we shall explore physiological solutions that the animals have found to the challenges of living in shifting-sand deserts, and the potential consequences for the conservation of the animals of their special physiology. Many of those solutions have been revealed by field studies because, while laboratory studies can simulate simple challenges like dehydration, they cannot simulate the mosaic of simultaneous challenges that will be encountered by animals in a shifting-sand desert (Costa & Sinervo, 2004). Laboratory studies reveal what animals can do, but not what they will do (Fuller *et al.*, 2014). In this first of the companion reviews, we give an overview of the communities of the animals of shifting-sand deserts and their physiological challenges, how they undertake the arduous task of locomotion on and in loose desert sand, and address the hypothesis that they would prefer to be on the sand surface but have to retreat into the sand because the surface is too hot, too cold, too desiccating or too dangerous.

While species like camels and the Arabian oryx are iconic of deserts, our review will focus on animals that spend some or all of their lives in, rather than on, the sand. That criterion means that we have to exclude the birds of the sandy deserts, even though there are conservation lessons to be learnt from those desert birds.

## A home in the sand

**Figure 1:**
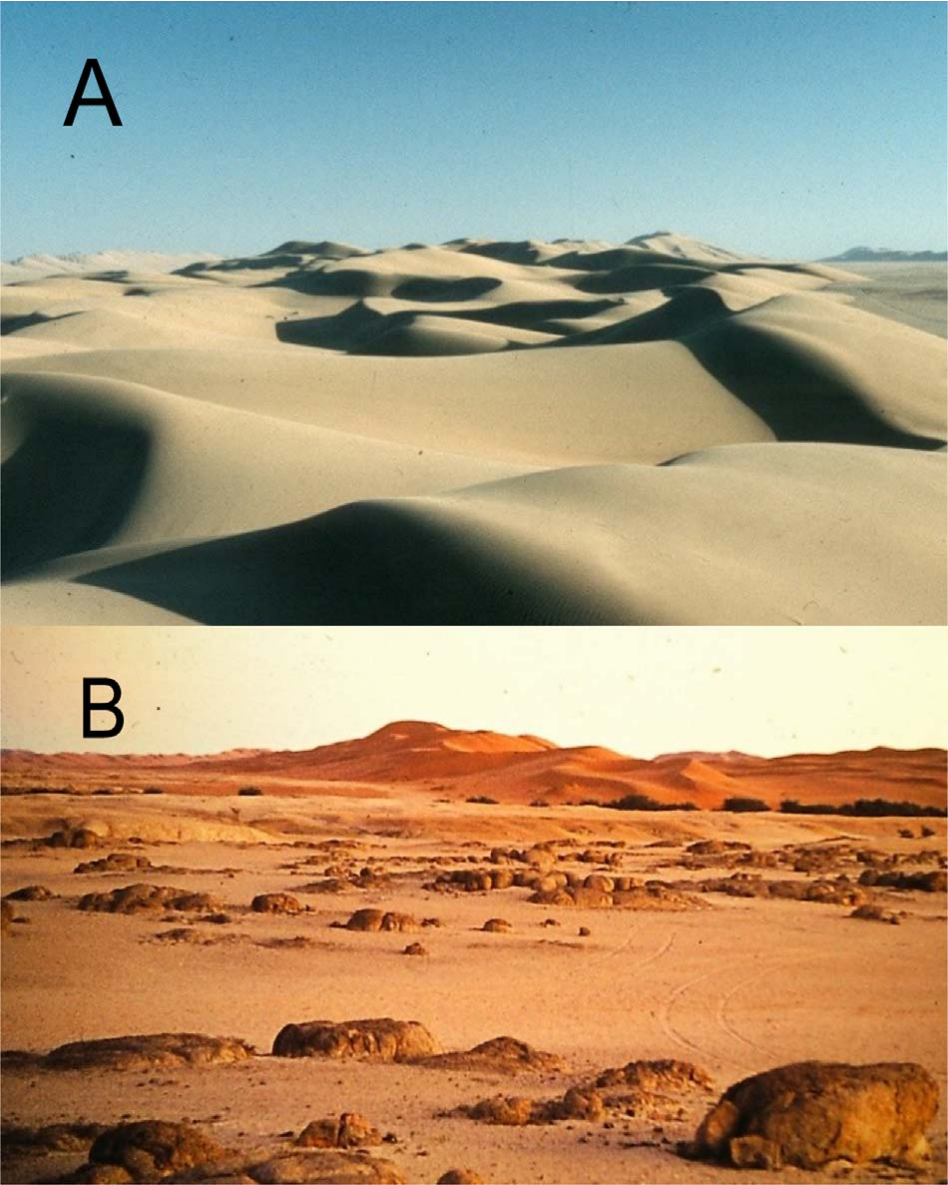
Dune fields of (**A**) the central dune sea, Namib Desert, Namibia and (**B**) the Simpson Desert, Australia. Photos: Duncan Mitchell, copyright retained.

From a distance, shifting-sand deserts appear devoid of animal life and so appear irrelevant to the conservation physiology of animals (see Fig. 1). But they are neither devoid of animal life nor irrelevant (Seely, 1984; Walsberg, 2000; Robinson & Barrows, 2013). A century ago, Vernon Bailey, Chief Field Naturalist of the US Department of Agriculture, remarked that the fact ‘that our driest deserts are abundantly populated with animal life has caused much surprise and speculation’ (Bailey, 1923, p. 66). The vast majority of their animals are invertebrates that live permanently below the sand surface and are invisible from a distance. Perhaps the most abundant invertebrates of shifting-sand deserts are the wingless insects *Zygentoma* (silverfish), which are extraordinarily xerophilic (Edney, 1971a; Zúñiga-Reinoso & Reinhard, 2019), though poorly studied, probably because they are hard to see (Smith, 2017). In shifting-sand deserts, *Zygentoma* will be a ubiquitous food source for insectivores and omnivores (Lawrence, 1959; Crawford, 1979; Watson & Irish, 1988), although Crawford (1979) and Hadley and Szarek (1981) considered nematodes to be more abundant than are *Zygentoma*. The larvae of tenebrionid beetles also will not be obvious to an observer because they live below the sand. Their adults are active on the surface of all shifting-sand deserts and are the most conspicuous of the diurnal surface-active animals of the Namib Desert. According to Cloudsley-Thompson (2001), viewing the Namib, ‘not infrequently, the only animal to be seen during the day, if any is visible at all, will be a tenebrionid beetle’. Termites are an important resource for the provision of both energy and water to desert insectivores and omnivores (Huey *et al.*, 1974; Crawford, 1979; Murray *et al.*, 2016; see Mitchell *et al.*, 2026) and are scarcely visible from a distance, even when they are on the surface. Though termites live in cavity nests in firm habitats, they forage on sand dunes, where they are major consumers of dune vegetative matter (Crawford, 1979).

That the Namib tenebrionids are visible in a desert during the day is unusual. The tenebrionid beetles of the Australian Simpson Desert are all active nocturnally (Duncan & Dickman, 2009), as are most tenebrionids elsewhere. While ancestral adesmine tenebrionids were active diurnally, there have been at least four phylogenetic shifts to nocturnal or crepuscular activity (Swichtenberg *et al.*, 2023). That their inhabitants are often nocturnally active is another reason for the false impression that shifting-sand deserts are devoid of animal life, because casual observers usually survey those deserts only during the day.

Insects and nematodes are not the only invertebrate inhabitants of shifting-sand deserts. They share the sands with arachnids, especially mites and spiders (Hadley & Szarek, 1981), and not always amicably. Spiders seem to be the only animals that can construct a burrow in the shifting sand. Most spider species achieve that engineering feat by using silk to line the inner wall of the burrow as they excavate it (Lubin & Henschel, 1990; Henschel *et al.*, 1995; Henschel, 1997; Albín *et al.*, 2016; Foelix *et al.*, 2017). The silk does not provide structural strength but aligns the adjacent sand particles, as a stonemason might do (Foelix *et al.*, 2017). Not all of the spiders of the shifting-sand deserts construct burrows, though. In the Namib sand sea, at least three species swim in the sand (Henschel, 1997).

It is perhaps not remarkable that small invertebrates, like silverfish and spiders, can acquire enough oxygen to swim in the loose sand of shifting-sand deserts because they have a low demand for oxygen. But, remarkably, vertebrates, including mammals, can do so. A 20- to 35-g mammal, the Namib golden mole *Eremitalpa granti namibensis* can swim more than half a metre below the surface of the sand (Holm, 1971, Fig. 2). Golden moles can breathe, move, rest and feed while buried (Gasc *et al.*, 1986), but the moles forage, well after dusk, fully emerged on the sand surface. They can travel more than 4 km in a night (Holm, 1971), though 20–1400 m is more typical (Fielden *et al.*, 1992; Seymour *et al.*, 1998). Indeed, most, but not all, of the vertebrates of shifting-sand deserts that can swim in the sand prefer the surface for routine locomotion.

**Figure 2:**
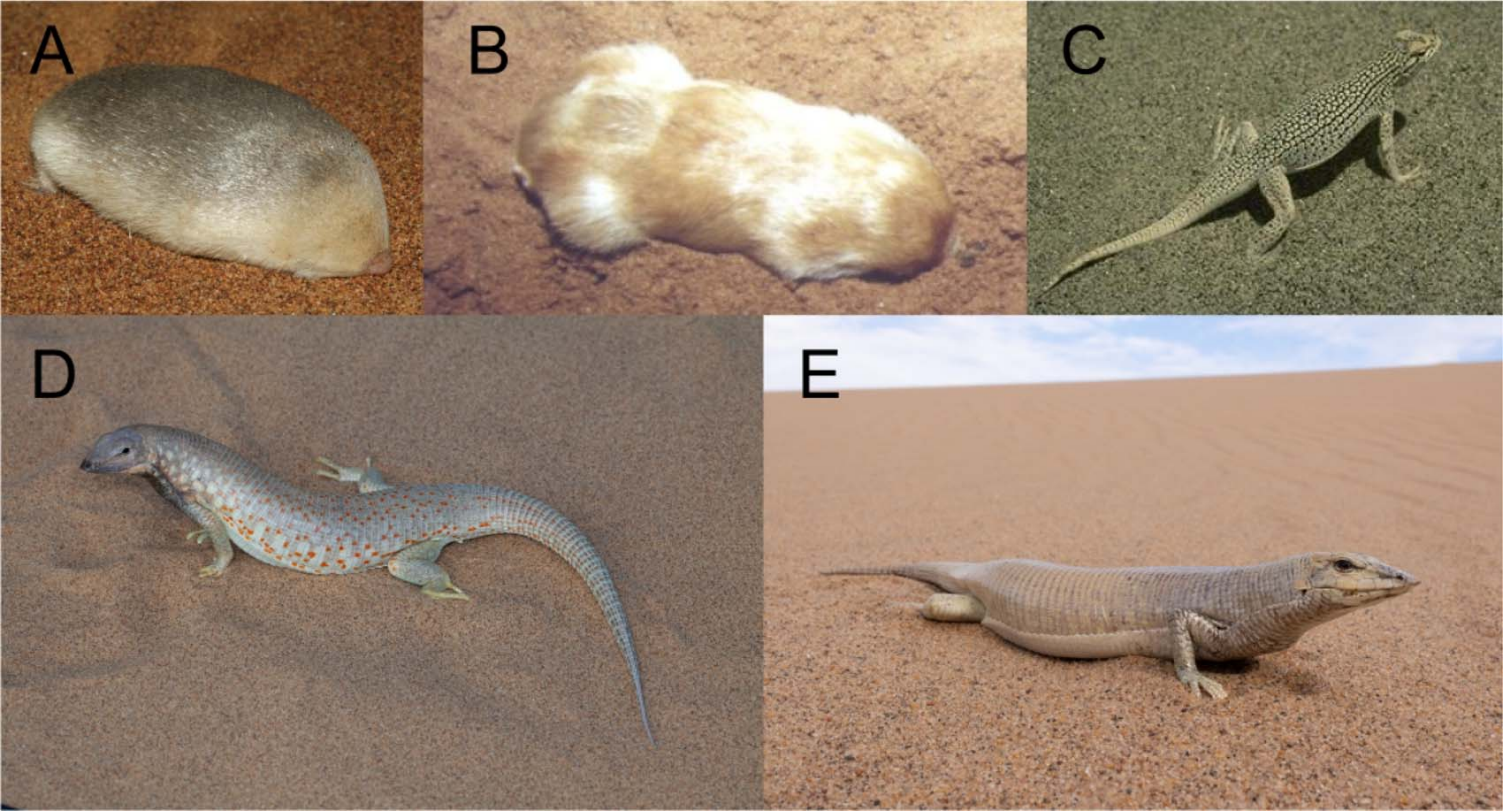
Some of the vertebrates of shifting-sand deserts. (**A**) Namib golden mole *E. granti namibensis*, Namibia*.* Photo: Joh Henschel, copyright retained. (**B**) Marsupial mole *N. caurinus*, Australia*.* Photo: Kyle Armstrong, copyright retained. (**C**) Coachella Valley fringe-toed lizard *U. inornata*, North America. Photo: Public domain: https://commons.wikimedia.org/wiki/Category:Uma_inornata. (**D** and **E**) Desert plated lizard *G. skoogi* (male D, female E), Namibia. Photo: Johan Marais (African Snakebite Institute), copyright retained.

Mammals, like the golden moles, are not the most common vertebrates in the shifting-sand deserts of the world; lizards are (Louw & Holm, 1972; Pianka, 1985; Higham, 2015). According to Robinson and Barrows (2013), there are 11 genera of sand-swimming lizards on four continents. Though some larger reptiles, like the snake *Eryx conicus*, engage in ‘quiescent burial’ (see below), the largest true sand-swimming animal of which we are aware is the desert plated lizard *Gerrhosaurus* (formerly *Angolosaurus*) *skoogi*, of the northern Namib Desert dune sea (Fig. 2D and E). The mass of those lizards can exceed 100 g, and snout-vent length can be longer than 150 mm. After an escape plunge into the sand, the lizards typically bury to 300-mm depth and then can move more than 500 mm laterally, presumably to deceive predators (Mitchell *et al.*, 1987). Like the golden moles, though, to get from place to place, the lizards prefer to use the sand surface.

Lizards that are adapted to desert sands often have snowshoe-like foot structures with fringed toes (Bauer & Russell, 1991) or even, in the case of *Pachydactylus* (formerly *Palmatogecko*) *rangei*, webbed feet (Lawrence, 1959; Higham, 2015). Toe fringes have evolved at least 26 times in seven different families of lizard (Luke, 1986). When the fringes were removed from the feet of the Mojave fringe-toed lizard *Uma scoparia*, their top speed on sand from the Mojave Desert was about 22% lower than when the fringes were intact (Carothers, 1986). When lizards run on the sand, they can run at near-maximum speed, even if they are undisturbed (Jayne & Ellis, 1998; Higham, 2015), regardless of the incline (Irschick & Jayne, 1999). Sand-burying lizards from the South American *Liolaemus* clade ran faster on loose sand than they did on simulated substrates of rock or bark, but so did species from the same clade that live in habitats with firm ground or rock (Tulli *et al.*, 2012). The observation implies that the ability to move rapidly on sand is not a special adaptation of the desert dwellers, an observation that is inconsistent with the ‘home ground advantage’ hypothesis (Zheng *et al.*, 2024). In perceived danger, some lizards will run to protective vegetation if it is available (Mosauer, 1935; Attum *et al.*, 2007; Kacoliris *et al.*, 2010; Evans *et al.,* 2017), while other species dive into the sand, either where they are or on a nearby dune slipface (Halloy *et al.*, 1998; Evans *et al.,* 2017).

In the North America deserts (Brown, 1973; Bauer & Russell, 1991; Eisenberg, 1975), and perhaps the Thar Desert of India (Goyal & Ghosh, 1993), the diversity of rodent species can exceed that of lizards. Those deserts are much younger than the deserts where lizards predominate (Pough *et al.*, 1978). The aeolian sands of the Mojave Desert seem to be less than 10 000 years old (Clarke, 1994); those of the Namib Desert are more than 50 million years old (Lawrence, 1959; Ward *et al.*, 1983). In those younger deserts, rodents will visit areas of loose sand to forage, to seek mates or just in transit, but they are not residents of the shifting-sand areas. Rodent burrows have to be constructed in firm or compacted soil, where a soil biocrust is available, or where loose sand has been stabilised by rain (Perrin & Boyer, 1996; Akin *et al.,* 2023) or by antelope urine (Seely, 1977). Thus, desert rodents do not have a refuge at their feet. Undoubtedly, there are conservation lessons to be learnt from desert rodents, but they are beyond the scope of our review here.

A burrow can serve many purposes, as a retreat from heat, cold or wind, a refuge from predation, a site for birth and care of young, a home for commensals, an exploration route for food such as tubers, a place to cache food, a toilet area or a site for sleep and torpor (see Kinlaw, 1999). Some residents of burrows never travel more than one body length from the mouth of the burrow (Contreras & McNab, 1990). However, the contention that ‘the possession of a cool, moist burrow with stable temperatures underground is especially critical for survival in arid zones that are hot and dry with greatly fluctuating temperatures on the surface’ (Kinlaw, 1999, p. 127) is not a general rule, because sand-swimming animals survive in shifting-sand deserts without a burrow*.* The ultimate purposes of sand swimming, without a burrow, are more limited, and may be confined to safety from surface threats that can be abiotic, like solar radiation or cold nights, or biotic, like predation or intraspecific aggression (Ryberg & Fitzgerald, 2015). Often, the main purpose of sand-swimming is taken to be escape from hostile conditions on the surface, as expressed by Dawson *et al.* (1989): ‘Most desert animals survive because they avoid extreme conditions by exploiting favourable micro-climates within the desert ecosystem’. Almost never has the assumption that the sand is a favourable microclimate, less hostile than the surface, been explored. We shall explore the advantages and disadvantages of being in versus on the sand. Then, since some invertebrates and almost all vertebrates that live in shifting-sand deserts spend time on the surface, we need to explore how they cope with the surface conditions, and particularly the solar radiation that penetrates the clear desert skies, and, in some deserts, freezing winters.

## Swimming in the sand

Beloved by makers of nature films since Walt Disney is the captivating undulation (Mosauer, 1930; Astley *et al.*, 2020) of a snake as it sidewinds over desert sand. The movement is common to many desert snakes, for example, the sidewinder rattlesnake *Crotalus cerastes* of the northwestern American deserts (Secor *et al.*, 1992), the horned viper *C. cerastes* of the Sahara and Middle Eastern deserts (Dorfman *et al.*, 2023) and Peringuey’s adder *Bitis peringueyi* of the Namib Desert (Brain, 1960, Fig. 3). It is a misconception, however, that sidewinding is an adaptation to shifting-sand deserts (Cowles, 1956). That form of locomotion is used in any smooth open territory and is more efficient on other open substrates than it is on desert sand (Cowles, 1956; Astley *et al.*, 2020; Dorfman *et al.*, 2023). The horned viper *C. cerastes*, at least, abandons sidewinding when it moves quickly under threat; it adopts a head-up flight posture with only two points of body contact with the ground (Winser, 1989). What is unique to shifting-sand deserts is not the locomotion that is used on top of the sand, but the locomotion that is used within the sand. How do animals deal with locomotion within desert sand?

**Figure 3:**
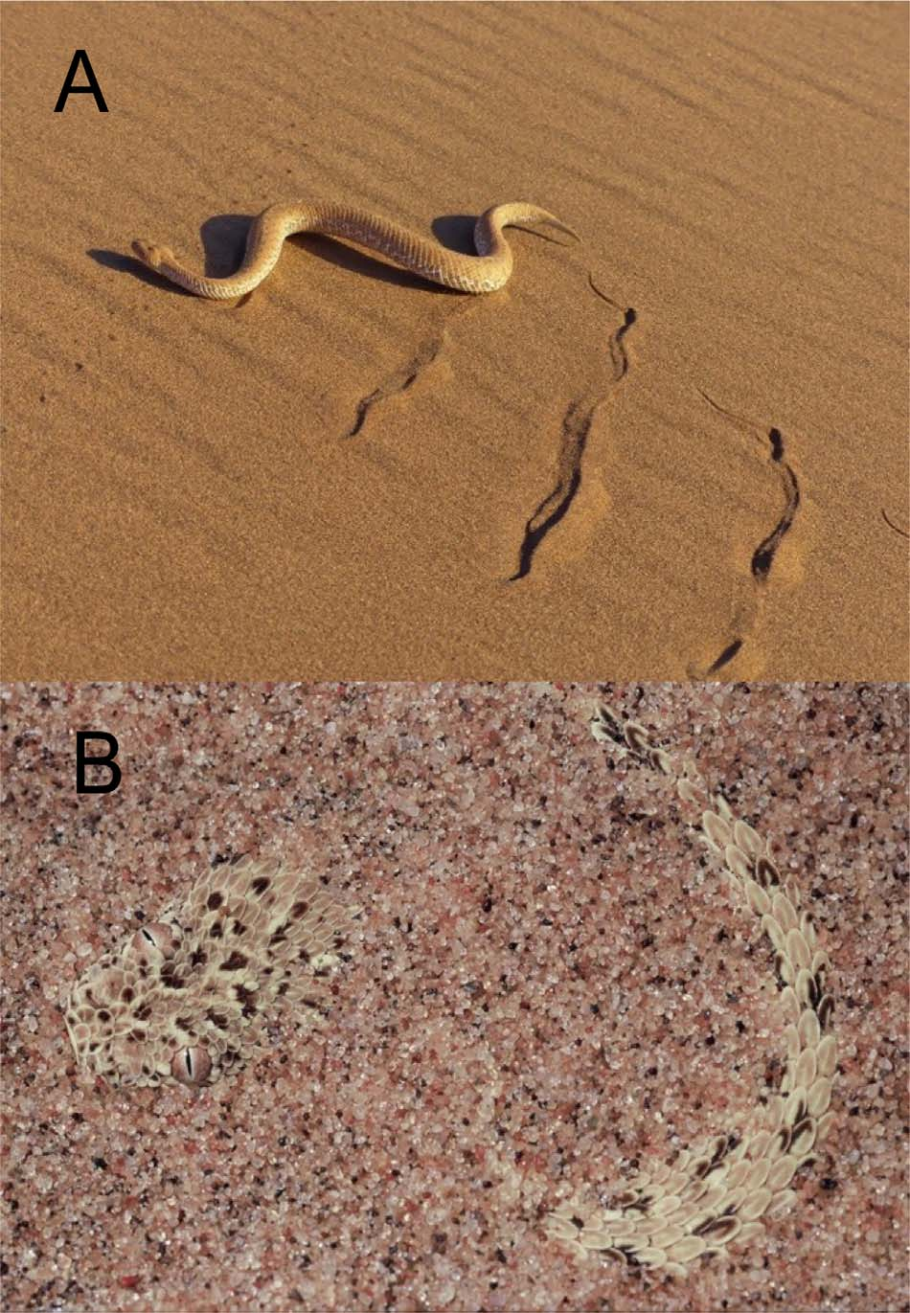
Peringuey’s adder *B. peringueyi* sidewinding (**A**) and after quiescent burial (**B**) in the sand of the Namib Desert. In panel B, the body is partially exposed but often just the head will protrude above the surface, or the entire snake can be completely buried and invisible, but just below the surface. Photos: Johan Marais (African Snakebite Institute), copyright retained.

We agree with Pough that true sand-swimming, as undertaken by golden moles or desert plated lizards, should be distinguished from what he calls ‘quiescent burial’, in which an animal embeds itself superficially in the sand surface. The animal can remain invisible if the surface is not disturbed and does not move more than a few body lengths in the sand (Pough, 1970), except perhaps for males when they seek mating opportunities (Henschel, 1990). Quiescent burial is employed by the fringe-toed lizards (*Uma* species) of the north-western American deserts (Stebbins, 1944) and many snake species (see Fig. 3). The tiny (<1 g) skink *Lerista labialis,* which inhabits the crests of sand dunes in the Simpson Desert, Australia, typically is found within 20 mm of the surface (Greenville & Dickman, 2009). In the laboratory, 20 mm also was the typical burial depth of the sand-swimming ocellated skink *Chalcides ocellatus*, of peri-Mediterranean deserts (and other habitats) (Catena & Hembree, 2014). The flat-tail horned lizard *Phrynosoma mcallii* of the Sonoran Desert uses quiescent burial between intermittent diurnal spells of activity but can sand-swim to depths of 100 mm overnight (Heath, 1965).

**Figure 4:**
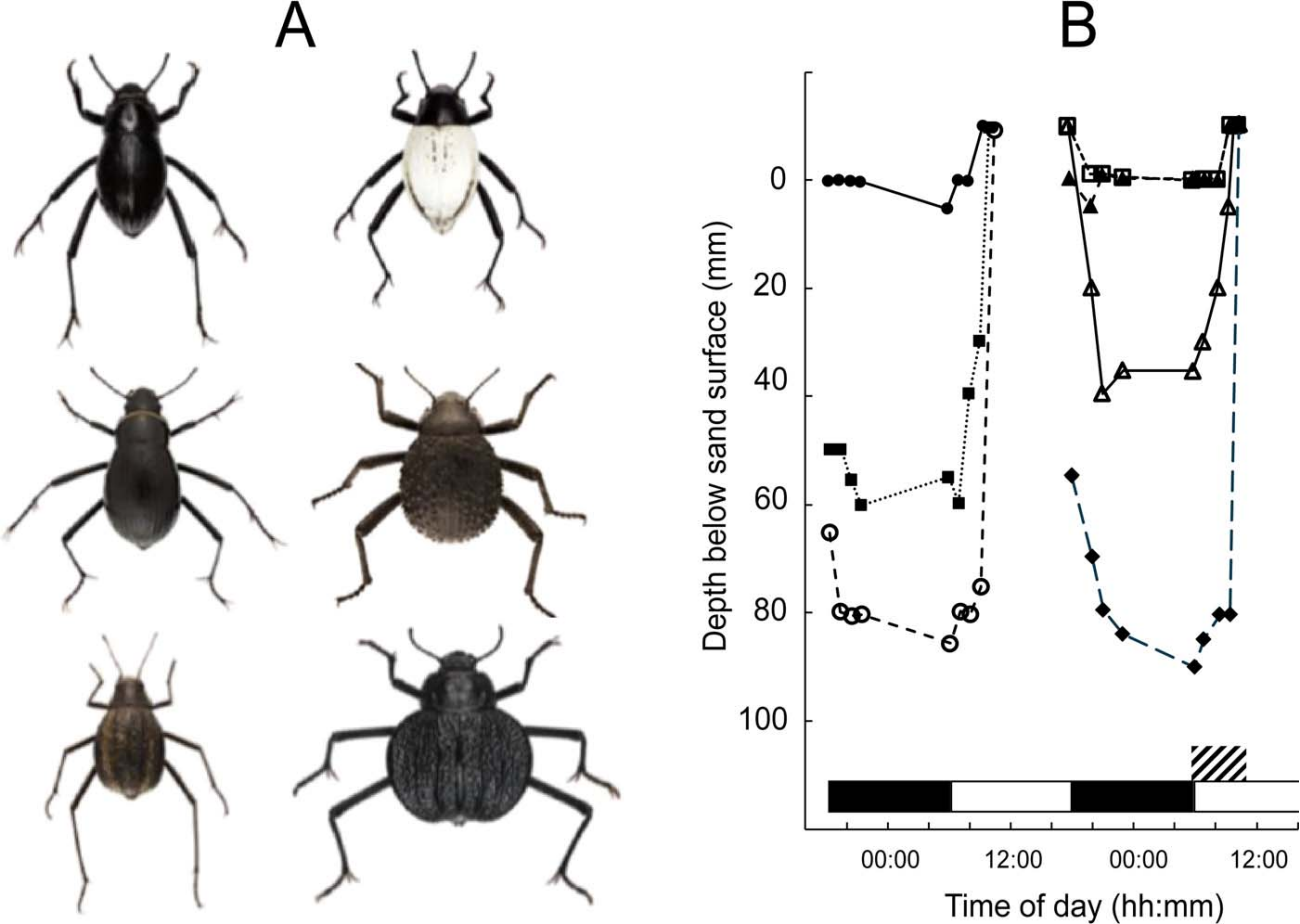
(**A**) Some sand-swimming tenebrionid beetles of the Namib Desert. Top left to bottom right: *Onymacris unguicularis, Onymacris bicolor, Onymacris laeviceps, Physosterna cribipes, Stenocara gracilipes, O. plana.* Composite by Trip Lamb (East Carolina University, USA). Reconstructed from Mitchell *et al.* (2020), by courtesy of Trip Lamb. (**B**) Movements of seven *O. unguicularis* beetles in the sand of a Namib Desert sand dune across 2 days. Each symbol and line is one individual. During the night, each beetle maintained a fixed distance from the surface, which differed between beetles, and they all emerged rapidly after sunrise (open bars on the *x*-axis indicate daytime, and closed bars on the x-axis indicate nighttime), whether or not there was fog (hatched bar). Iridium-192 wires (4–6 mm, 0.3-mm diameter) were glued to the carapace, and gamma radiation was detected at the sand surface. Measure of radiation attenuation allowed depth to be estimated to an accuracy of 10 mm. Redrawn from Seely *et al.* (1985).

The way that quiescent buriers bury can be explored simply by watching them (Benesch & Withers, 2002), but locomotion within the deep sand cannot be seen and so requires special techniques, most of which can be implemented only in the laboratory (Hosoi & Goldman, 2015). In what we believe to be the only tracking of the unimpeded movements of animals in the sand in their natural desert habitat, we used iridium-192 wire tags to track tenebrionid beetles in the sand of a Namib Desert sand dune (Fig. 4; Seely *et al.*, 1985). Earlier, a different isotope had been used by Edney *et al.* (1978) to track the subsurface movements of desert cockroaches in the laboratory, as had Kenagy (1973) to estimate the depth of rodents in burrows in the field.

Swimming in the sand requires a special relationship between the sand and a swimming animal, a relationship with profound consequences for conservation because it implies that the animals cannot exist elsewhere. No more could a Namib golden mole survive on a savannah than could a whale survive there. ‘Organism and environment form an inseparable pair’ (Bartholomew, 1964, pp. 8–9). Animals of the shifting dunes of the Namib Desert are absent from the adjacent riverine sand and gravel plains (Seely & Louw, 1980). In the shifting-sand deserts, the sand is comprised of solid granules that have to be displaced around the swimming animal. The term ‘like a fluid’ is used frequently (Medvedev, 1965; Shimada *et al.*, 2009; Wu *et al.*, 2015) but is not really accurate because, in a fluid, the forces that resist the passage of a solid object are viscous forces, that is, frictional forces that are generated when adjacent layers of fluid flow over one another (Goldman, 2014). Contra Baumgartner *et al.* (2008), the forces that are generated between the flowing sand granules are repulsive contact forces (Maladen *et al.*, 2009, 2011; Ding *et al.*, 2012), and, unlike viscous forces, those repulsive contact forces are independent of the speed of movement of the animal through the sand (Astley *et al.*, 2020), at least at biologically relevant speeds (Bergmann & Berry, 2021). When it has been measured, the physiology supports the physics of sand swimming; the intensity of activity of locomotor muscle of the sandfish *Scincus scincus*, a sand-swimming lizard that is native to north Africa and the Arabian Peninsula, is independent of its speed of movement through sand (Sharpe *et al.*, 2013). The drag force also is independent of volume fraction, the ratio of material volume to total occupied volume, and therefore independent of how much air is in the sand (Maladen *et al.*, 2009). At a site in a fluid, pressure is independent of direction, but the lateral forces on an animal that is buried in the sand are lower than the vertical force (Norris & Kavanau, 1966). However, as in a fluid, the drag force on the animal is dependent on the size of the animal and on the depth at which the animal is moving (Ding *et al.*, 2012; Peng *et al.*, 2016). Again the physiology supports the physics: the intensity of activity of locomotor muscle that is required to achieve the same movement by a sandfish increased with depth in the sand (Sharpe *et al.*, 2013).

In most solid substrates, the drag forces would render swimming too expensive as a locomotor alternative. For the drag forces to be sufficiently low to make sand-swimming a viable locomotor alternative requires that the sand granules be large for a substrate, and the granules of desert sand are about 1000 times larger than the particles that make up silt and clay, in which sand-swimming is impossible (Brown, 1978). The drag is also lower if the granules are ‘approximately spherical’ (Astley *et al.*, 2020); spherical glass beads offer a lower resistance to movement than do natural substrates that have the same granule volume (Bergmann & Berry, 2021). Animals tend to avoid swimming in sand that has a granule diameter bigger than 2 mm because swimming in granules that are too large is exhausting (Stebbins, 1944; Pough, 1970).

Roundness is a property of aeolian sands that is derived from a complex interaction between wind speed (and other wind features) and the size of sand granules (Thomas, 1987). As we shall see, the roundness creates enough space between granules to permit the ready diffusion of gases, and so allows sand-swimming animals to breathe (Mitchell *et al.*, 2026). The roundness of sand granules tends to be consistent within a desert, but differs between deserts (Goudie & Watson, 1981). The granules of older deserts tend to be rounder than are the granules of younger deserts, but the granules of the Namib Desert, the oldest desert (Ward *et al.*, 1983), are not as round as might be expected from its antiquity (Goudie & Watson, 1981). Consequently, the percentage of the bulk volume of sand that is occupied by interstices (porosity) in Namib sand is high, but not as high as might have been expected (Dickinson & Ward, 1994).

The morphology of the animals that swim in those sand granules varies vastly (see Fig. 2). The animals vary in size, from *Zygentoma* at about 20 mm long to the lizard *G. skoogi* with a snout-vent length of 150 mm. Their bodies may be rigid, as in the adults of the tenebrionid beetles, or flexible, as in the larvae of the same beetles. They may be elongated, like the legless *Acontias* (formerly *Typhlosaurus*) skinks or *Chionactis* snakes, or almost oblate spheroids, like the golden moles. They can have eight legs (spiders) or no legs (snakes, legless lizards). They can be without hair (lizards and snakes) or furred (golden moles and marsupial moles). Yet convergent evolution has bestowed on them some common features, including a streamlined body, a snout or other rostral structure that is suitable for insertion into sand, an abrasion-resistant surface, protected sense organs and mechanisms that keep sand out of body cavities.

Nothing is known (at least to us) about how *Zygentoma* or other soft-bodied invertebrates move in or on the sand. The cost of transport has been measured for soft-bodied fly larvae when they crawled in a glass running tube and the results implied that limbless soft-bodied invertebrates might incur the highest cost of surface locomotion of all animals (Berrigan & Lighton, 1993). Adult tenebrionids that sand-swim tend to be smaller than are other tenebrionid species, and the swimmers have leg tarsi that seem ideally shaped to sweep back sand (Medvedev, 1965). Typical of the sand-swimming beetles is ‘a streamlined and ‘drop-shaped’ body, the pronotum merging smoothly with the elytra, a small head withdrawn into the thorax’ (Coineau *et al.*, 1982). According to Cloudsley-Thompson (2001), the selection pressures that have resulted in the shape of desert tenebrionids, such as the widening and flattening of the body, relate more to the requirements of movement in the sand than they do to thermal or hygric stresses.

**Figure 5:**
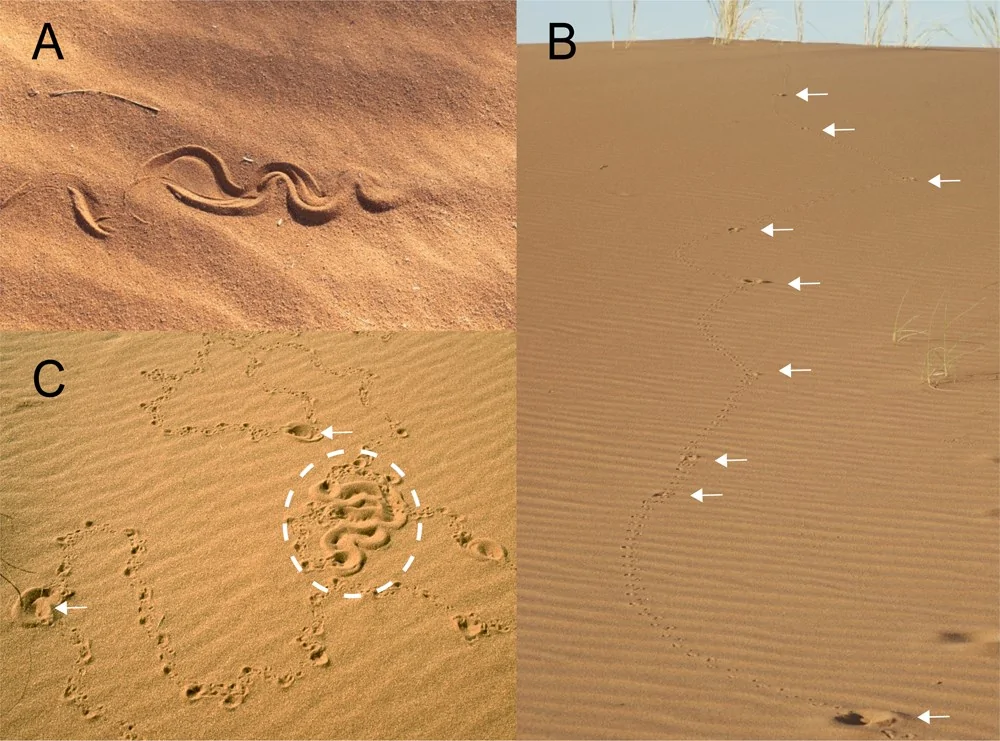
Tracks in the Namib Desert sand. (**A**) An unidentified lizard swimming just below the surface from left to right of the photo. Photo: Johan Marais (African Snakebite Institute), copyright retained. (**B**) Namib golden mole *E. granti namibensis* foraging on the surface, heading for *Stipagrostis* grass tufts on dune crest. Note head-dipping sites, indicated by white arrows, thought to be where the mole makes seismic measurements. Photo: Joh Henschel, copyright retained. (**C**) Non-linear foraging by a Namib golden mole on the surface, with frequent head-dipping sites (arrows), and then burial that threw up sinusoidal crests (within the white circle), presumably at an encounter with buried prey. Photo: Roger Seymour, copyright retained.

The rigid body of a beetle precludes the undulatory movement that is ubiquitous in sand-swimming reptiles. Those reptiles use lateral deformation waves along the body (Peng *et al.*, 2016), in one plane (see Fig. 5A), to propel themselves forwards, a movement that is portrayed as ‘slithering’ (Astley *et al.*, 2020) or ‘writhing’ (Mitchell *et al.*, 1987). Bauer and Russell (1991) described the movement as ‘low-amplitude, high-frequency, lateral undulations’, and Maladen *et al.* (2011) described it as ‘single-period travelling sinusoidal wave down its body’. Chong *et al.* (2022) described (and modelled) it as ‘a linear combination of standing wave body bending and traveling wave body undulation’*.* There is no lateral displacement of the centre of mass of the body (Norris & Kavanau, 1966). For a more detailed description of the mechanics of both sand-swimming and quiescent burial, please see Appendix 1 in the supplementary material.

The sand-swimming reptiles tend not to use their limbs to propel themselves through the sand (see Appendix 1). So, not surprisingly, many sand swimming reptiles have reduced legs and sometimes no legs. By contrast, the forelegs of sand-swimming mammals are well developed and clawed, and are used to propel those mammals (Gasc *et al.*, 1986), normally through the sand but through semolina in the studies of Gasc and colleagues. The best studied of the sand-swimming mammals is the Namib golden mole *E. granti namibensis* (Meester, 1964, Fig. 2), but we do not agree with Gasc *et al.* (1986) that the golden mole ‘is the only truly desert-adapted fossorial insectivore’. In a striking example of convergent evolution of physiology, morphology and ecology (Withers *et al.*, 2000; Feigin, 2025), the marsupial moles *Notoryctes typhlops* and *Notoryctes caurinus* swim in the sand of the deserts of the central western interior of Australia and eat insects, along with other food, as do golden moles (Holm, 1971). With their oblate spheroid shape (see Fig. 2), 30-g mass, smooth fur, absent external ears and vestigial eyes, with a cloaca, and internal testes in the males (the only testicond marsupials), the marsupial moles look remarkably like the Namib golden mole. It takes inspection of a female marsupial mole in hand to discover its pouch, facing backward where it does not act as a scoop in the sand. Unlike the Namib golden mole, though, neither of the marsupial mole species emerges to forage; both of the species spend virtually their entire lives in the sand where they are active at least 600 mm below the sand surface (Pavey *et al.*, 2012).

Though it can forage successfully on unvegetated sand (Fig. 5C), the Namib golden mole relies on clumps of desert grasses to improve foraging efficiency. When they are on the surface, the golden moles often travel in remarkably straight lines between those clumps (Lewis *et al.*, 2006; see Fig. 5B). The attraction is not the grass itself but the invertebrate prey that congregates at the roots, particularly termites. In their dune habitat, food resources are poor and patchy, and the moles must seek congregated prey, if they can (Fielden *et al.*, 1990a). With vestigial eyes that cannot form images, the moles cannot see the grass clumps, but they have enlarged ossicles that are suitable for the detection of low-frequency vibrations in the sand (Lewis *et al.*, 2006). The moles seem to detect the grass using seismic cues. In wind, the grass clumps generate vibrations that are transmitted through the sand. When the moles travel on the surface, they stop and dip their heads into the sand every 600 mm or so, presumably to detect those vibrations (Lewis *et al.*, 2006; see Fig. 5B). The moles also may detect vibrations that are generated by the movement of the termites, or other buried invertebrates, themselves (Fielden *et al.*, 1990a; Mitchell & Seely, 1994). Figure 5C shows the tracks of a mole that apparently discovered prey on an open dune.

Though the Australian marsupial moles do not forage on the surface, they probably also use seismic cues to detect their animal prey. The sandy plains on which they live are sparsely vegetated with mallee shrubs and spinifex grass (Pearson & Turner, 2000), and invertebrates congregate in the spinifex hummocks (Ward, 2016). The moles are true sand swimmers and not burrowers, and they can swim, in the laboratory, for 40 min at speeds of up to 18 m·h^−1^ (Withers *et al.*, 2000). Other than that, little or nothing is known about how marsupial moles move in the sand, what causes them to move in a particular direction or why they use costly underground foraging rather than foraging on the surface as does the Namib golden mole. The same question can be asked of the other 19 species of golden mole, since only the Namib golden mole and the giant golden mole *Chrysospalax trevelyani* forage on the surface (Fielden *et al.*, 1990a).

## Coping with the heat and cold in and on the sand

On clear days, the surface temperature of the sand has reached 70°C in the Namib Desert (Marsh, 1985a) and Australian deserts (Sommer & Wehner, 2012) and 60°C in the Colorado Desert (Mosauer, 1935). No animal in contact with a surface at those temperatures would survive more than for a few minutes. Some animals of shifting-sand deserts that ought to be capable of activity on the surface, like the Australian marsupial mole (Pavey *et al.*, 2012), seldom emerge on to the surface, whatever the temperature. Others emerge regularly. One hypothesis for why some emerge regularly in warm conditions is that they seek a body temperature that is favourable for themselves or for the symbionts that help to digest their food. We will argue against that hypothesis because those animals usually can attain any body temperature without leaving the sand (Seely, 1979). As far as we know, no desert animal that is beetle size or larger can seek mates in the sand, and so mating is a compelling reason to emerge. Many species obtain some or all of their food on or above the sand surface (see Mitchell *et al.*, 2026). Others have to emerge to access water, especially the droplets that are formed by fog or dew (see Mitchell *et al.*, 2020).

One way to avoid dangerous heat stress during activity on the sand surface would be to confine surface activity to the night, as do all the tenebrionid beetle species of the Australian deserts, and many tenebrionid species in deserts elsewhere (Duncan & Dickman, 2009; Bartholomew & El Moghrabi, 2018). Why is nocturnal activity not universal in shifting-sand deserts? Maybe the nights are too cold. In some deserts, the insects have developed freeze resistance (Cloudsley-Thompson, 2001). Maybe the reason is that nocturnal predation is too high (Heath, 1962, 1964; Duncan & Dickman, 2009), though the leopard ctenotus *Ctenotus pantherinus,* a lizard of Australian arid areas, seems to be forced into nocturnal activity because avian predators are diurnal (Gordon *et al.*, 2010).

In biomes other than deserts, vertebrates that are active in diurnal heat often can maintain a thermal steady state by evaporative cooling (see Mitchell *et al.*, 2018). The desert probably creates a selection pressure against the use of body water for evaporative cooling as a routine strategy (Cowles & Bogert, 1944; see Mitchell *et al.*, 2026). Some desert lizards do pant, but only as a last resort, at body temperatures that are highly correlated with their critical thermal maxima (Bradshaw, 1988) and close to their lethal limits (Heath, 1965; Loughran & Wolf, 2020). Rather than use water, the animals of shifting-sand deserts rely mostly on behavioural mechanisms to thermoregulate. They share with the animals of other biomes a drive to seek shade (if it is available) or to change posture or orientation (Stevenson, 1985). A thermoregulatory behaviour that is available to them, but not to the animals of other biomes, is to enter the sand that is beneath their feet at any time.

### Life in the thermal boundary layer of shifting-sand deserts

**Figure 6:**
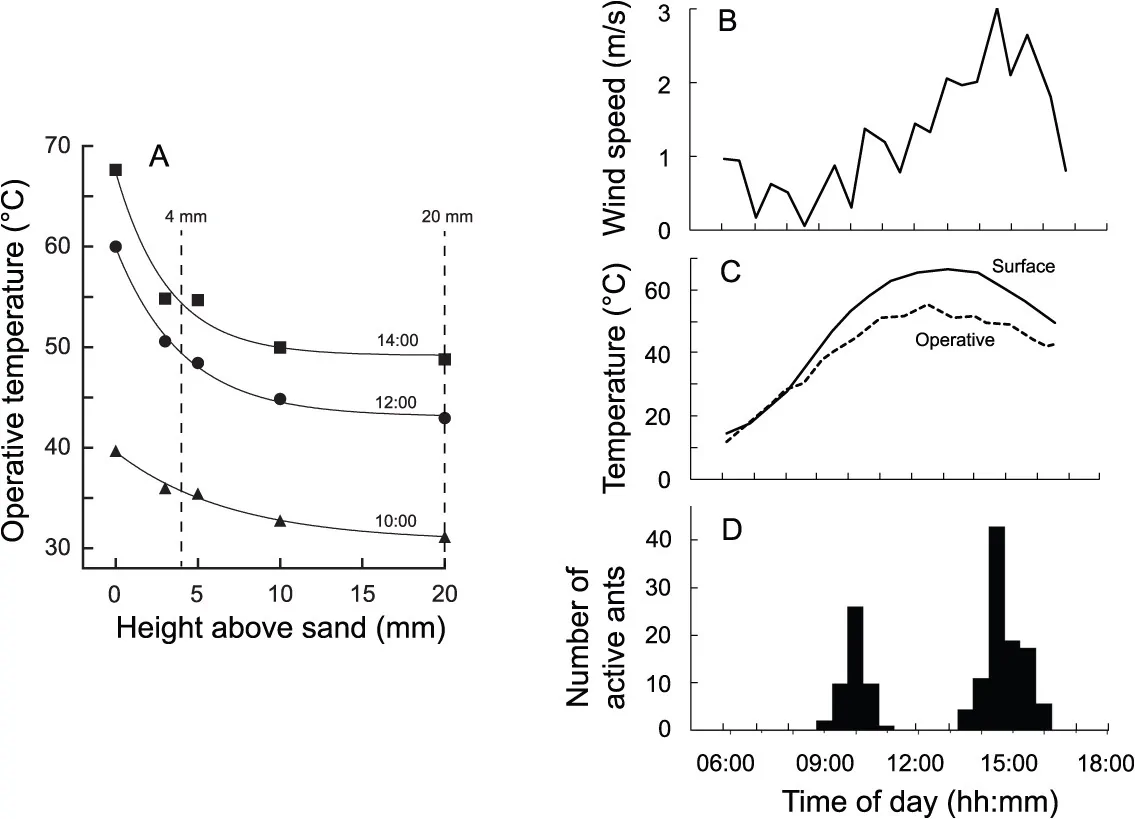
(**A**) Operative temperature in the air boundary layer used by *O. robustior* ants in the Namib Desert, on an autumn day. Operative temperatures were measured with a thermocouple (0.08-mm diameter) inserted into the thorax of a dead ant (mean mass, 4 ± 1 mg) and hand-held above the surface. The live ants typically held their thorax about 4 mm above the ground when they ran. (**B**–**D**) Microclimate at the height of *O. robustior* ants running on the sand surface of the Namib Desert, near a nest, and (D) the number of ants that returned to the nest in 10-min periods, over that autumn day (C). (B) Wind speed measured with a hot-wire anemometer at an elevation of 4 mm. (C) Surface temperature and operative temperature at ant height. Redrawn from Marsh (1985a) by permission of the University of Chicago Press. At the time, *O. robustior* was not separated from *Ocymyrmex barbiger* (Marsh, 1986).

On the surface of a hot shifting-sand desert, to cope with the heat of the day essentially means to cope with solar radiation, direct and indirect. An animal has to deal with the radiation that impacts directly on it, which can be directly from the sun itself or any of the reflected or diffracted components of that radiation (Mitchell *et al.*, 2024), as well as the indirect consequences when the sun heats the substrate. Extended diurnal activity on the surface of a shifting-sand desert is possible only in those deserts where the air is cool, such as the Namib Desert. In the Namib, the prevailing wind is an onshore wind that blows in from the adjacent cold upwelling of the southern Atlantic Ocean. The interaction between that relatively cool air and the hot sand surface creates a thermal boundary layer with sharp changes in air temperature close to the ground (Porter *et al.*, 1973). Even just a few millimetres above the surface, the microclimate is much cooler than it is on the surface itself (Fig. 6A). When surface temperature was close to 70°C, the operative temperature at the elevation that is typical of a running *Ocymyrmex robustior* ant was 15°C lower.

The critical thermal maximum of *O. robustior* ants is about 52°C (Marsh, 1985a). The behavioural activity of the ants is thermophilic; the peak of their activity in the afternoon occurred at an operative temperature that was within a few degrees Celsius of that critical thermal maximum (Fig. 6). But the surface activity was distinctly bimodal because the ants retreated into the sand when the environment became unacceptably hot around solar noon, and re-emerged when the sand had cooled. The same operative temperature existed on the sand surface at 10:00 and 14:30, but there were more ants active in the afternoon than in the morning (Fig. 6D). The higher afternoon activity was not because the higher wind speed in the afternoon (Fig. 6B) offered more convective cooling; the operative temperature takes account of the rate of convective cooling. The bimodal activity pattern (Ward & Seely, 1996; Robinson & Barrows, 2013), and the phenomenon of continuing surface activity until retreat is compulsory (at least by invertebrates) both seem to be characteristic of the activity patterns of many species that are diurnally active on hot days in shifting-sand deserts. In cooler seasons, or if the thermal environment remains tolerable in hot seasons, the pattern of surface activity typically is unimodal, so unimodal activity appears to be the preferred mode.

Burial into sand was not the only thermal retreat that was available to ants. Even though the air at 4 mm above the sand surface was 15°C lower than the surface temperature, the microclimate would still have been too hot for the ants to be active and safe. When they were on the surface, they sought brief periods of respite (mean duration 5 s, Marsh, 1985a) by climbing on to any irregularity on the sand surface. That climb elevated them typically to 10–20 mm above the surface. As Fig. 6A shows, at 14:00, when the ants started to re-emerge, the operative temperature at running height (4 mm) was above their critical thermal maximum, but the conditions at an elevation of 10 mm were below their critical thermal maximum. Climbing onto local irregularities as a means of increasing convective cooling is a common behaviour of invertebrates of deserts that have a source of cool air (Henschel *et al.*, 2001).

**Figure 7:**
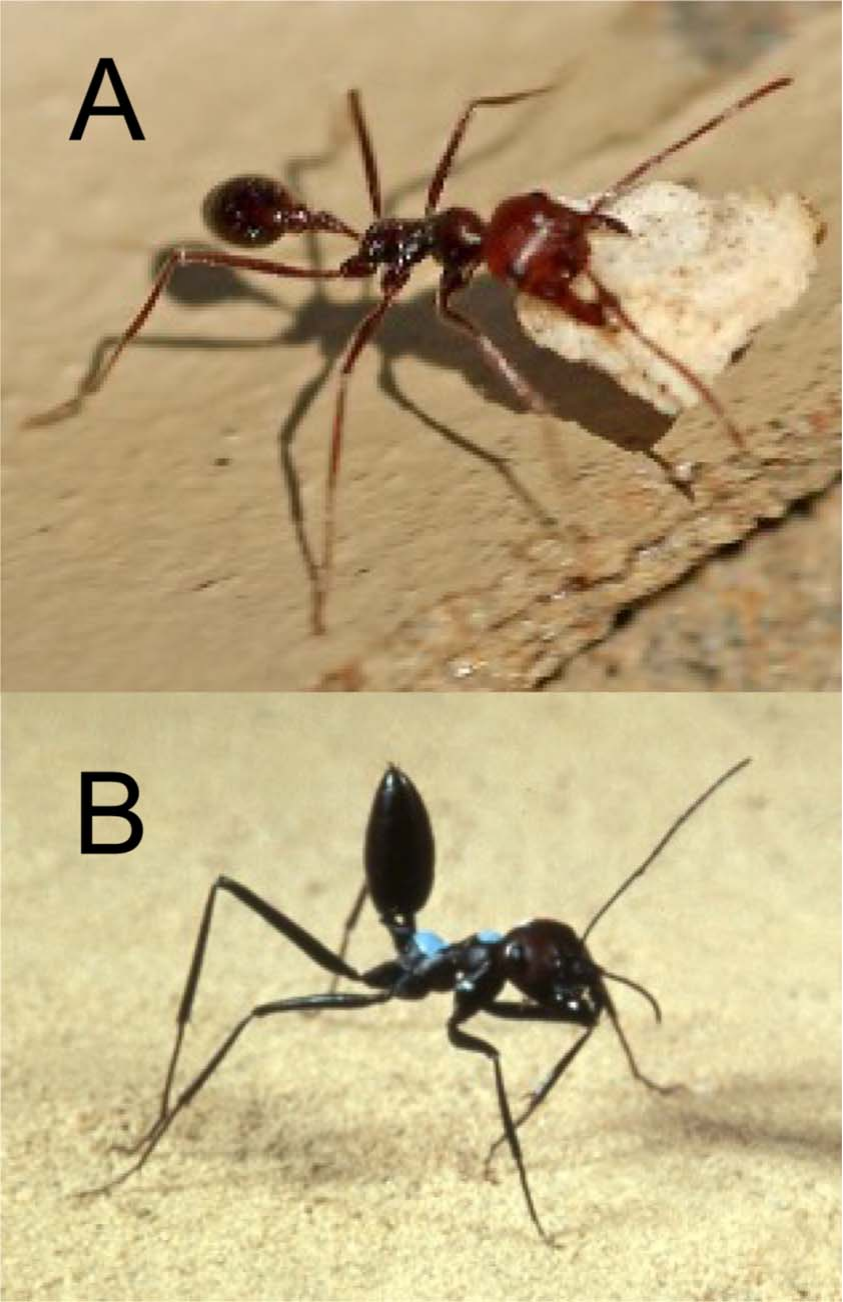
(**A**) *Ocymyrmex robustior* from the Namib Desert. Photo: Joh Henschel, copyright retained. (**B**) *Cataglyphis bicolor* from the Sahara Desert, with paint dots for identification. Photo: Rüdiger Wehner, copyright retained. From Wehner (2020): Desert navigator*. The Journey of an Ant.* Harvard University Press, Cambridge, USA.

The ability of the ants to tolerate heat is aided by their morphology (Fig. 7). They may be the most dolichomorphic (long and slender) of all animals with legs. Their shape allows them to present little area to the sun and other sources of radiant heat, while creating a very high convective heat transfer coefficient (Mitchell, 1974). There is an advantage to having the morphology and behaviour that allows the ants to be active on a hot desert surface. The ants feed on the carcasses of invertebrates that do not have those adaptations, have misjudged the danger from the surface heat and have succumbed (Marsh, 1985b).

In the Sahara Desert, the Saharan silver ant *Cataglyphis bombycina* and Sahara Desert ant *Cataglyphis bicolor* (Fig. 7B) occupy a niche that is identical to that of *O. robustior* in the Namib Desert. *Cataglyphis* is phylogenetically distant from *Ocymyrmex* (Wehner & Wehner, 2011), but the genera have evolved very similar morphology, including long legs (Sommer & Wehner, 2012). The *Cataglyphis* species also have a diet and behaviour similar to that of *O. robustior*, refined for their circumstances. *Cataglyphis bombycina* has a critical thermal maximum of 54°C (Wehner *et al.*, 1992), exceeded marginally by *C. bicolor*, at 55°C (Gehring & Wehner, 1995). In both *O. robustior* and *C. bombycina*, foraging is done by sterile older workers, reducing the consequences for the colony of a lethal accident to a forager (Wehner *et al.*, 1992).

Even though the critical thermal maximum of *C. bombycina* exceeds that of *O. robustior*, the activity period of individual *C. bombycina* lasts only 3.6 ± 0.6 min (Wehner *et al.*, 1992), about one-tenth of the activity period of individual *O. robustior* (Marsh, 1985b). The daily activity period for the entire foraging force of a *C. bombycina* colony can be shorter than 20 min (Wehner *et al.*, 1992). Further, even those short periods of activity are mostly spent in elevated respites (Wehner *et al.*, 1992). *Cataglyphis bombycina* seems to have been forced into extreme thermophilia and compressed surface activity because of voracious predation by Duméril’s fringe-fingered lizard *Acanthodactylus dumerilii*. The lizards inhabit burrows in the vegetated edges of Saharan sand dunes. The ants are the sole prey of the lizard, and, even though they have much larger thermal inertia, the lizards are not quite as tolerant of heat as the ants (Wehner *et al.*, 1992). Foraging ants do not emerge until scouts from the nest have detected that the lizards have been forced to retreat to their burrows by the heat (Wehner & Wehner, 2011).

Ants of the *Cataglyphis* genus also are found in the Arabian, Taklamakan and Gobi deserts (Wehner & Wehner, 2011). A third genus of thermophilic ant, *Melophorus*, occurs in Australia (Sommer & Wehner, 2012). *Melophorus bagoti* is even more tolerant of heat than are the *Cataglyphis* species. Its critical thermal maximum is 57°C and it achieves peak surface activity at substrate temperatures of 60°C (Christian & Morton, 1992). Perhaps uniquely amongst thermophilic ants, *M. bagoti* has an aversion to ‘lower’ environmental temperatures. *Melophorus bagoti* usually does not emerge until the surface temperature exceeds 50°C (Wehner & Wehner, 2011). *Melophorus bagoti* occupies arid zones in Australia but does not appear to occur in shifting-sand deserts. That may be because Australian shifting-sand deserts do not benefit from the cool onshore winds that generate a favourable boundary layer.

Beetles potentially benefit more from the thermal boundary layer than do ants. The diurnally active tenebrionid beetles of shifting-sand deserts run with their head, thorax and abdomen about 15 mm above the sand surface (Roberts *et al.*, 2025), meaning that they have access to cooler parts of the boundary layer than do ants. Not all tenebrionid beetles have long legs (Medvedev, 1965), but the adesmine tenebrionids that inhabit sand dunes do have legs that are long enough to raise their other body parts above the surface (Cloudsley-Thompson, 2001; Ward, 2016). There has been much conjecture, and some experimental evidence, that those long-legged beetles respond to high surface temperature by ‘stilting’, that is increasing their elevation further, which seems like a plausible scenario. But when body temperature actually was measured, stilting did not reduce it appreciably (Ward & Seely, 1996). Inspection of Fig. 6A reveals that a shift from 15 to 20 mm would produce little extra cooling. What does decrease body temperature appreciably, at least in the fast-running Namib tenebrionid *Onymacris plana*, is running in the boundary layer. The additional convective cooling removes more heat than is created by the metabolism of exercise and by solar radiation (Roberts *et al.*, 2025). *Onymacris plana* is the first, and so far, the only, terrestrial animal demonstrated to cool by exercising.

As with the ants, the physiology of those tenebrionids that have evolved to be active diurnally in some shifting-sand deserts includes tolerance of high body temperature. Although body temperatures of up to 50°C have been recorded in individual beetles (Roberts *et al.*, 1991), the mean body temperatures of six species of Namib adesmine beetles on the surface in the field was 29.9 ± 4.2°C for *Physadesmia globosa* (which inhabits dry shifting-sand riverbeds) and 38.8 ± 3.8°C for the sand dune specialist *O. plana* (Seely *et al.*, 1988a). On average, those body temperatures were just 0.6 ± 0.3°C lower than the concurrent black globe temperatures in their respective microclimates (Seely *et al.*, 1988a). By contrast, the body temperature of the diurnally active tenebrionid *Adesmia cancellata* of the Kuwaiti desert was 20–24°C (Abushama & Al-Salameen, 1989). Two species of *Eleodes* had temperatures of just 16–18°C in a Californian desert, at the air temperatures of 12–28°C at which they were active on the surface (Kramm & Kramm, 1972).

The principal behavioural response to the thermal environment that is used by the animals of shifting-sand deserts is an adjustment of activity period (Stevenson, 1985). Beyond that response, the beetles of shifting-sand deserts seem to rely much more on microclimate selection than they do on postural change as a mechanism for the behavioural alteration of body temperature (Ward & Seely, 1996). The lizards of shifting-sand deserts, on the other hand, can and do use both postural change and microclimate selection. The primary postural change that they use is to orient their bodies parallel to or transverse to the sun, which alters the surface area on which direct solar radiation is incident (Heath, 1965). Horned lizards *Phrynosoma* sp. can also change the shape of their body and increase or decrease the area that is exposed to direct sunlight (Heath, 1965). Most desert lizards are taller than are ants or beetles, so lizards are exposed to even cooler parts of the boundary layer. The lizards of hot shifting-sand deserts, such as the wedge-snouted skink *Chalcides sepsoides* of the North African and Middle Eastern deserts (Attum *et al.*, 2007), can have disproportionately long legs. Some lizards also use postural changes that enhance convective cooling in the boundary layer during hot parts of the day. The famous ‘thermal dance’ (Louw & Holm, 1972; https://www.youtube.com/shorts/qszdh0ha0OU) of the Namib dune lizard *Meroles anchietae*, and the elevation of the hind feet of *G. skoogi* (Seely *et al.*, 1988b; see Fig. 2D), in our view, have nothing to do with the sand being too hot for their feet, as surmised originally by Mosauer (1935) and endorsed by Hamilton and Coetzee (1969). Rather, they are postural changes that introduce their fine digits, with high convective heat transfer coefficients (Mitchell, 1974), into a layer of cooler and faster-moving air, a proposal that assumes that the digits are perfused well enough to transport heat. When *G. skoogi* lizards were placed momentarily on sand at a temperature that is painful to humans, they dorsiflexed their backs and lifted all four feet simultaneously, apparently removing sensitive body parts and leaving only a small area of belly in contact with the sand (Seely *et al.*, 1988b). The zebra-tailed lizard *Callisaurus draconoides* is a 10- to 20-g thermophilic iguanid of the deserts of the western USA and of Mexico. Though the lizard is typically encountered on shifting-sand expanses in the desert, it is not a sand swimmer but retreats to shallow burrows in sand that is stabilised by plants (Tanner & Krogh, 1975). *Callisaurus draconoides* can counter the warming effects of solar radiation in hot parts of the day by elevating its body above the sand surface and so enhancing convective cooling (Muth, 1977).

While lizards do bury in the sand or implement postural changes that enhance heat transfer, their preferred means to prevent overheating is to adjust their activity period, or to seek shade, if it is available, or to change location on sand dunes. There is strong seasonal variability in the activity periods of desert lizards (Huey *et al.*, 2021b). The horned lizards *Phrynosoma platyrhinos* and *P. mcallii*, of the Mojave and Sonoran deserts, are capable of burying in sand but prefer habitats where they can reach the shade of shrubs (Heath, 1965). But some lizards of shifting-sand deserts live on vegetationless dunes, or dunes where vegetation is so sparse that shade is inadequate. As an example of how they cope, *G. skoogi* extended their activity period on the surface by choosing areas that enhanced warming in the early morning and late evening, and delayed or obviated the need to retreat into the sand in the hottest part of the day by choosing areas that enhanced cooling (Mitchell *et al.*, 1987).

The diurnally active lizards and arthropods of shifting-sand deserts spend their nights buried in the sand, and inactive. That raises questions about the cues that they use to time their emergence on to the surface. The animals use either an endogenous cue or some cue of which we humans are unaware. The cue cannot be a thermal cue. Many are so deep in the sand that they could not possibly sense the thermal conditions on the sand surface (see Section Is the desert sand a thermal refuge or a thermal haven? below). It is likely that the cues come from endogenous clocks. The timing of activity by an endogenous clock allows animals to emerge at or before the time that surface conditions are appropriate for activity, and test the conditions in the boundary layer. In terraria, 40% of horned lizards *Phrynosoma* sp., from the Mojave and Sonoran Deserts, emerged before dawn (Heath, 1962, 1965). The timing of emergence by endogenous clocks has been proposed for other lizards (Gordon *et al.*, 2010) and for tenebrionid beetles (Cloudsley-Thompson, 2001). While the molecular basis of the circadian clock is well characterized as a transcriptional translational feedback loop (Huang, 2018), the mechanism(s) that underlie the timing of ultradian events is/are less well understood (Goh *et al.*, 2019). The functioning of the clocks that determine emergence of the animals of shifting-sand deserts has received little attention. Norris and Kavanau (1966, p. 662) proposed that the shovel-nosed snake *Chionactis occipitalis* ‘becomes active spontaneously 24 h or less after the initiation of its last activity period’ in the Sonoran and surrounding deserts.

We have contended that the timing of animal activity on the surface of hot shifting-sand deserts relies on the nature of the thermal boundary layer, as well as access to shade, if available. Our observations of the surface activity of the lizard *G. skoogi* on Namib sand dunes (Seely *et al.*, 1990) have revealed that the microclimate of the boundary layer there was less extreme and more stable during the activity period than could have been predicted from either air temperature or sand surface temperature. The thermal mosaic of microclimate across the dunes was much richer than would have been expected from sand surface temperatures. The measuring instruments that we used were too big to properly resolve the microstructure of the boundary layer. We have proposed miniature globe thermometers and ultrasonic anemometers as better instruments with which to measure microclimates (Mitchell *et al.*, 2024). It is likely that a full understanding of the thermal physiology of the surface activity of the animals of shifting-sand deserts will depend on proper measurements of the microclimate in the thermal boundary layer.

### On being black in hot shifting-sand deserts

That most of the adult tenebrionid beetles that are active diurnally on the surface of hot shifting-sand deserts had black carapaces was as astounding to the biologists who first studied them (Hamilton, 1973; Cloudsley-Thompson, 1979) as were the observations that desert ravens were black and that the nomadic Bedouins of the deserts of the Middle East wore black cloaks. We now understand the value of that black outer layer for the interception of solar radiation by the ravens and by the Bedouin cloaks. The ravens re-radiate the heat while their body is protected by the insulation of the feathers (Marder, 1973), and up-drafts under the Bedouin cloak dissipate the radiant heat by convection and evaporation (McCance *et al.*, 1974; Shkolnik *et al.*, 1980). There are black phenotypes of the sand-swimming legless lizard *Anniella pulchra* of Californian deserts, but they are confined to moister coastal sand and the lizard is not active on the surface under strong solar radiation (Klauber, 1932; Miller, 1943). We are far less sure of the physiology behind the black colour of adult tenebrionid beetles. Being black, rather than a lighter colour, would allow the beetles to warm up more rapidly in the morning sun and so reduce the period of sluggishness after morning emergence (Lovegrove, 1995). What else we know is summarised in Appendix 2. Irrespective of what the advantages are, we advise conservationists not to assume that a black animal in a hot shifting-sand desert necessarily is alien or compromised.

Long before Hamilton (1973) pondered the consequences of being black for Namib tenebrionid beetles, Cowles and Bogert (1944) had drawn attention to the propensity of desert lizards to function, apparently voluntarily, at body temperatures that are close to their critical thermal maxima, although subsequent measurements showed that the body temperatures of active diurnal desert lizards always were at least 6°C lower than their critical thermal maxima (Huey, 1982). However, it was the observation that black tenebrionid beetles maintained the highest known body temperatures of any ectotherm when they were active diurnally on the sand dunes of the Namib Desert (Edney, 1971b) that prompted Hamilton to advance his theory of ‘maxithermy’. He proposed that the high body temperatures were beneficial because they increased the rate of biological processes and were physiologically possible because the beetles always had access to a thermal refuge. They could retreat into the sand below their feet before their body temperature became dangerously high (Hamilton, 1973). He postulated, but did not prove experimentally, what the processes that benefitted from high body temperature might be: digestion by symbionts, assimilation of food or unspecified reproductive processes. Hamilton’s maxithermy hypothesis has been subsumed into a more general paradigm that holds that ‘hotter is better’ (Angilletta *et al.*, 2010).

Not long after Hamilton proposed maxithermy, Heinrich (1977) advanced an alternative explanation for the high body temperatures of the beetles. He proposed that the primary goal of the beetles’ thermoregulatory behaviour was to achieve a stable body temperature, rather than a target body temperature, and that stability was easier to achieve at high than low body temperature. Neither theory has received lasting support. A fundamental problem with both theories is that, while most desert tenebrionid beetles are black, only the Namib tenebrionid beetles have such high temperatures (Seely *et al.*, 1988a). Why not tenebrionids elsewhere? That question remains unanswered. If Hamilton was right, Ward (1991) expected even those Namib tenebrionids to operate at a lower body temperature if food was limited, and they did not do so. If maxithermy was beneficial for the digestion and absorption of food (Bartholomew & El Moghrabi, 2018), then one might expect to find maxithermy in other ectotherms that are sympatric with the beetles, and it is not present in all Namib lizards (Mitchell *et al.*, 1987; Pietruszka, 1988). If Heinrich was right, why did the Namib beetles not evolve the biochemistry so as to function nocturnally, when the management of a stable body temperature becomes easier without the problem of solar radiation (Ward, 1991)?

### Is the desert sand a thermal refuge or a thermal haven?

**Figure 8:**
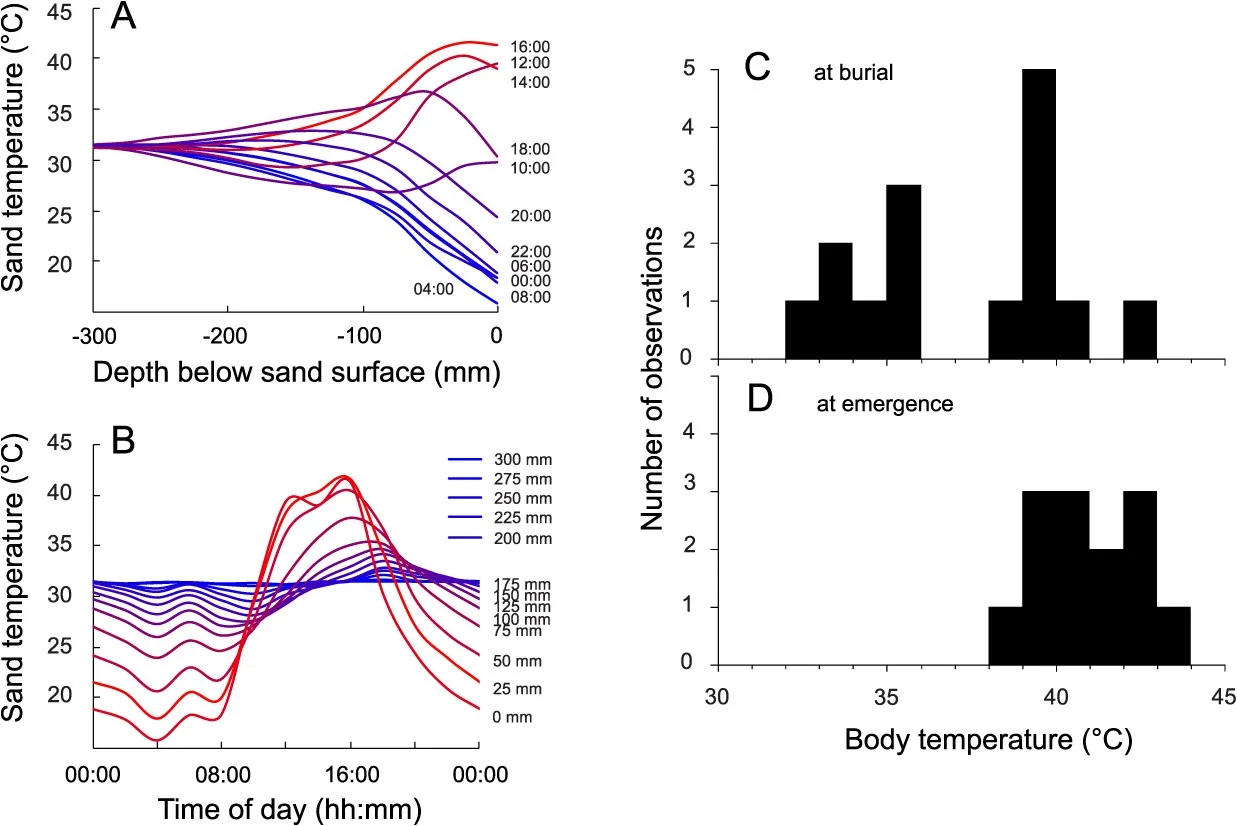
(**A** and **B**) Temperatures on and below the sand surface of a Namib Desert sand dune, at two-hourly intervals, on a spring day, as a function of depth below sand surface (A) and local time of day. (B) Temperatures were measured with an array of nine thermocouples attached to a thermally inert rod. Reconstructed from Seely and Mitchell (1987). (**C** and **D**) Body temperatures of *U. inornata* lizards in a gradually heated terrarium that simulated the deserts of California, USA, as they buried when sand temperature rose (C) and then re-emerged (D) as sand temperature rose further. Figure originally published in *Copeia* by Pough (1970: Fig. 4) and redrawn here with permission from the American Society of Ichthyologists and Herpetologists (ASIH) and by courtesy of the author.

Figure 8 shows how the temperature of the sand changed with time and depth below the surface, on a spring day in the Namib Desert. The profile will be typical of the sand in other hot sunny deserts and has some notable features. First, even though the surface temperature was not nearly as high as it can get in the Namib Desert (Marsh, 1985a), the surface temperature varied by more than 25°C over the 24 h. By contrast, the temperature of the sand at 300-mm depth varied hardly at all. That attenuation of surface oscillations in temperature can be quantified in terms of the ‘damping depth’, which is the depth below the surface at which the amplitude of the oscillation in sand temperature is 1/e (about 37%) of the amplitude at the sand surface (Nobel & Geller, 1987). The damping depth was about 100 mm in the Sonoran Desert (Nobel & Geller, 1987) and was similar in the Namib Desert (Fig. 8A). There can be variation in temperature at depth with season (Seely & Mitchell, 1987; Huey *et al.*, 2021a). According to Porter *et al.* (1973), at 600 mm below the surface in desert sand, the temperature is close to the average dry-bulb temperature of the air over a month. The mean annual temperature at that depth currently varies little from year to year (Bennett *et al.*, 1988) but may increase with global warming.

Secondly, the highest sand temperature during a day was in the early afternoon and was not on the sand surface, but just below it (see Fig. 8A). That means that buried animals that are about to engage in an afternoon period of surface activity (see Fig. 6), presumably driven by a circadian clock, penetrate a barrier that is warmer than the surface before they reach the cooler boundary layer. Thirdly, at the typical time of emergence for a morning period of surface activity (see Fig. 6), the sand temperature did not vary much with depth; the animals would not have emerged along a thermal gradient. Lastly, at night and in the late afternoon, a buried animal could manipulate its microclimate substantially just by changing depth in the sand. That advantage would accrue to true sand swimmers, as it does to animals that use burrows in other deserts, but not to quiescent buriers, which would be subject to a wide range of sand temperatures, though they would escape the direct effects of solar radiation.

If there is an advantage for the biochemistry of an ectotherm to having a stable body temperature, like the presumed advantages of homeothermy in mammals and birds (Mitchell *et al.*, 2025), then an ectotherm that seeks that advantage on a typical day in the Namib Desert (as depicted in Fig. 8A) would have been served best by burying to 300 mm below the surface, or deeper. But ectotherms that need to be on the surface for at least part of the day or night, for example, for foraging, have to trade off the thermal benefits of deep burying against the curtailment of the period of surface activity by travel time to and from the surface (Huey *et al.*, 2021a). Any saving in travel time by staying close to the surface may be perilous, especially in deserts with cold winters. The body temperature of squamates that are too close to the surface can drop below their critical thermal minima, or even to freezing (Holm & Edney, 1973; Huey *et al.*, 2021a). To avoid freezing, the squamates of the deserts of north-west USA need to overwinter at least 200 mm below the sand surface (Huey *et al.*, 2021a). In a Californian desert, Cowles (1941) found that many squamates hibernated closer to the surface than that and argued that they tolerated high variability in substrate temperature, and even tolerated the risk of damage from cold in order not to miss out on surface activity if surface conditions became tolerable.

That the animals of shifting-sand deserts would prefer to be on the surface but cannot always be there because of the thermal challenge is a popular postulate (Stebbins, 1944; Dawson *et al.*, 1989), as we have said. So, the sand is a refuge. Pough (1970, p. 156) had no doubt that the sand provided a refuge, at least for the lizards of shifting-sand deserts: ‘burial is mainly a defensive device’. Pietruszka (1988, p. 175) agreed: ‘sand dunes provide unique habitats that may be simultaneously thermally intolerable and yet be the source of a virtually complete thermal refuge to diurnal ectothermic organisms’. The sand may be a refuge not only against cold night air or the consequences of intense solar radiation but also, and especially for invertebrates, against desert winds that are strong enough to move the surface sand (Seely & Mitchell, 1987). An alternate hypothesis is that the sand is a thermal haven for the animals, where especially the ectotherms would prefer to be, so that if they do emerge on to the surface, it is not to satisfy thermal requirements (Robinson & Seely, 1980; Seely, 1983). These postulates are not mutually exclusive, because, for the same animal, the sand could be a refuge in some circumstances and a haven in others. True sand swimmers can make use of the thermal mosaic that is offered in the sand at different times at different depths (Ward & Seely, 1996; Henschel *et al.*, 2001).

Arguing against the concept that the desert sand offered a haven that was sought out for its thermal benefits, Seely and Mitchell (1987) pointed out that Namib tenebrionid beetles could attain the body temperatures that they select in a thermal gradient, namely between 36 and 40°C, between 12:00 and 17:00 on the day depicted in Fig. 8, by burying to attainable depths, but that they chose to forsake the sand and to emerge onto the dune surface during that time. At 14:00, a time typical of afternoon emergence for Namib beetles and lizards on days when surface activity is bimodal, an animal buried on the day of Fig. 8 could find a microclimate with any temperature between 29 and 39°C simply by changing depth. So, the reason for emergence cannot be to seek an acceptable microclimate, though it could be to seek a better microclimate.

The sand is even less likely to offer a profitable thermal haven for those ectotherms of shifting-sand deserts that engage in quiescent burial, because those animals are confined to the first few centimetres of the sand surface. Figure 8B shows the thermal consequences of shallow burial for *Uma inornata* lizards of North American deserts. Using a ‘white infrared’ lamp over the sand in a terrarium, Pough (1970) simulated the effect of increasing solar intensity as a day progressed. The lizards buried, just under the sand surface, as the sand temperature increased, and then re-emerged with further increases in sand temperature. Consequently, their body temperature at emergence was higher than it was when they buried and exceeded 40°C in many individuals (Pough, 1970). Similarly, though shovel-nosed snakes *C. occipitalis* have been recovered at depths of 600 mm in North American deserts, where they may have been hibernating, they usually bury within 30 mm of the surface. At a depth of 30 mm, they must be subject to massive diurnal changes in temperature (Norris & Kavanau, 1966). For them, the desert sand that they choose is not a thermal haven.

**Figure 9:**
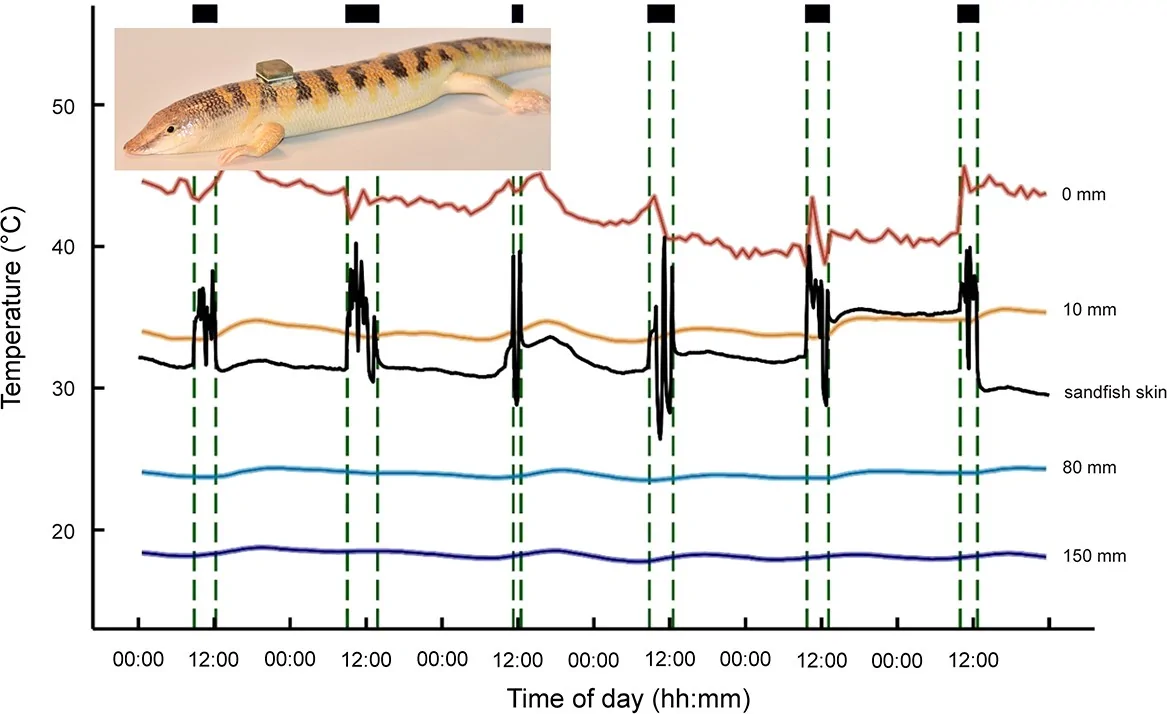
Skin temperature (black line) of a sandfish *S. scincus* in a terrarium (840 × 500 mm) that contained 150 mm of sand, with part of the terrarium surface heated from 08:30 to 16:00 by a ceramic lamp. The skin temperature of the sandfish was measured with a transmitting biologger that was glued above the forelegs (see insert); preliminary experiments showed that lizard surface temperature was close to cloacal temperature. The coloured lines show the temperature of the sand at different depths. The dashed vertical green lines enclose each day’s period of voluntary surface activity. Redrawn from Livingston *et al.* (2024), by courtesy of the BioOne Digital Library (https://bioone.org/) and of the authors, and by permission from *Journal of Herpetology.*

Until recently, it was a matter of conjecture how sand swimmers might use the thermal mosaic that is available to them in desert sand, because their temperatures had not been measured in the sand. Recently, the skin temperature of wild-caught sandfish *S. scincus* (Livingston *et al.*, 2024) was measured in a terrarium that contained 150 mm of builders’ sand, with part of the terrarium surface heated by a ceramic lamp. Swimming in builders’ sand would have been more costly than swimming in the rounder granules of desert sand. Figure 9 shows the temperature of one of the sandfish over a week. For most of the time, the sandfish had a stable skin temperature, near 32°C, presumably when it was inactive and buried more than 10 mm below the surface. The lizard never displayed a temperature that it would have attained if it had ventured as deep as 80 mm. During brief (about 4 h) daily periods of activity, its body temperature varied substantially, once by more than 10°C, and occasionally reached the temperature of the sand surface. Within those 4 hours, episodes of high body temperature were interspersed by episodes of lower temperature that implied that the sandfish had temporarily sought refuge deeper within the sand than when it was inactive. The body temperature of the sandfish remained well below the critical thermal maximum of 48.2 ± 0.5°C (*n* = 8) and even further above its critical thermal minimum of 10.6 ± 1.6°C (Livingston *et al.*, 2024).

### The poikilothermic mammals of shifting-sand deserts

There are mammals of shifting-sand deserts have forsaken homeothermy, usually considered a cardinal characteristic of mammalian physiology. As with the ectotherms of shifting-sand deserts, the body temperature of a burying mammal of a shifting-sand desert is determined by the temperature of its surroundings. The maintenance of homeothermy requires adequate food to maintain metabolic heat production in the cold and adequate water to provide evaporative cooling in the heat (Fuller *et al.*, 2026). Shifting-sand deserts are deserts because water is scarce, and they are poorly resourced with food because of that lack of water. Then, contact with the sand during burial will often impose high conductive heat transfer, and in sand that will normally be somewhat cooler than typical mammalian body temperature, resulting in an energy cost that is difficult to overcome for burying animals in a resource-poor environment (Stevenson, 1985).

**Figure 10:**
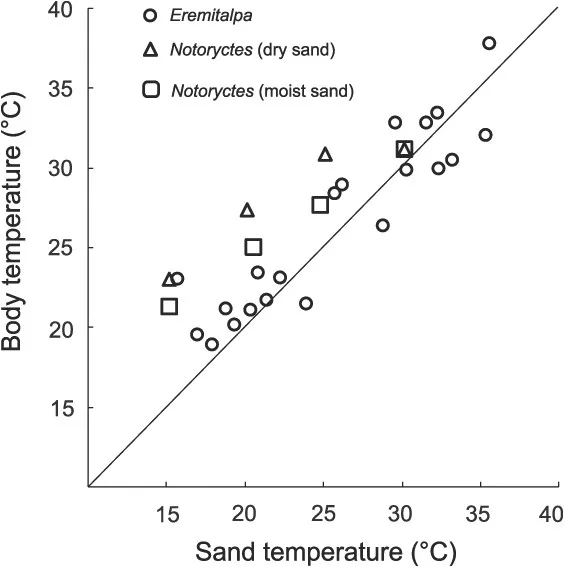
Cloacal temperature of 21 Namib golden moles *E. granti namibensis* immediately after they were captured on sand dunes in the Namib Desert, plotted against the temperature of the sand at the depth that they were captured, and cloacal temperature of a single marsupial mole *N. caurinus* at different sand temperatures in dry and wet sand in the laboratory. The line of identity is shown. Redrawn from Fielden *et al.* (1990b), by permission of Elsevier, with additional data from Withers *et al.* (2000).

Figure 10 shows the body temperatures of representatives of the two mammal genera that we have discussed (see Section Swimming in the sand). The body temperatures of Namib golden moles (*Eremitalpa*) were measured immediately (usually within 15 s) after their capture in the field (Fielden *et al.*, 1990b) and compared to the temperature of the sand at the depth that the moles were found. They were captured at depths from very close to the surface to 350 mm, and the temperature of the sand at their site of capture varied by more than 20°C. Over that range, the body temperature was close to that of the surrounding sand. There was no clustering of body temperatures around the body temperature of about 33°C that the golden moles selected (somewhat reluctantly) in the laboratory, so no evidence that free-ranging golden moles had selected a microclimate. At body temperatures below 20°C, the golden moles were uncoordinated but could dig in the sand. They became agitated at high body temperature; they have no effective mechanism of evaporative cooling (Fielden *et al.*, 1990b).

We can say less about the regulation of body temperature in the Australian marsupial moles (*Notoryctes*) because we have laboratory measurements only, and those were on one *N. caurinus* (Withers *et al.,* 2000). The mole never reached the temperatures around 37°C that are typical of mammals, and that the golden moles sometimes reached. The body temperature of the marsupial mole ranged from 23°C to 31°C over a range of air temperature from 15°C to 30°C. The data implied that the body temperature was not regulated but was dependent on the temperature of the sand around the mole (Fig. 10). McNab (1966) had concluded that all mammals that lived fully or partially below the ground would have a reduced capacity to regulate body temperature, but he would not have predicted that a mammal could be fully poikilothermic. Our mammals are not unique in that respect. The naked mole rat *Heterocephalus glaber*, a similar-sized burrowing rodent of African savannas, also is fully poikilothermic when it is away from its colony (Buffenstein & Yahav, 1991).

No one has investigated how the biochemical processes, and especially neurochemical processes, that have evolved to function optimally at around 37°C in homeothermic mammals have adapted to function in a poikilothermic mammal. But they have adapted; those poikilothermic mammals sense the environment, move, digest food and reproduce perfectly adequately at those poikilothermic body temperatures. But the moles cannot live anywhere other than in a shifting-sand desert.

### Facultative thermoregulation

Whereas the endothermic mammals of shifting-sand deserts appear not to regulate their body temperature at all, anomalously, some ectotherms of those deserts do seem to seek to regulate their body temperature, at least for some of the time. How important is selection of temperature in amongst the suite of potentially competing homeostatic processes that the animals may seek to implement? The two mammals of shifting-sand deserts (Fig. 10) clearly assign a low priority to the regulation of body temperature. There are examples of ectotherms that also assign a low priority to the regulation of body temperature. When the fog-basking tenebrionid beetle of the Namib Desert emerges to bask at night, it is at a body temperature that is far lower than they would entertain during their normal diurnal activity (see Mitchell *et al.*, 2020), and low enough to compromise their mobility seriously. So in that situation, they assign a much higher priority to the replenishment of body water than they do to the regulation of body temperature (Mitchell *et al.*, 2020). When it emerged from the sand, the sandfish in Fig. 9 allowed its body temperature to oscillate by 10°C, thus relaxing the stable temperature that it exhibited while buried, to engage in activities on the surface.

We and others believe that when the animals of shifting-sand deserts do seek to thermoregulate, thermoregulation always is facultative (Seely & Mitchell, 1987). The maintenance of a stable body temperature is never their highest priority, amongst homeostatic mechanisms. We have concluded that ‘if the lifestyle of *A. skoogi* were dictated by thermoregulatory demands, it would never emerge from the sand’ (Seely *et al.*, 1988b, p. 100). The general approach of the animal community of shifting-sand deserts to deal with heat and cold is to avoid body temperatures that are so high or so low that they are incompatible with other physiological functions, thus ‘providing a behavioral buffer from physiological failure’ (Livingston *et al.*, 2024, p. 383). Some of the animals emerge in the day, when they have to contend with heat that is compounded by solar radiation, and some at night, when heat loss to air and night sky might freeze them. The thermoregulatory processes that the animals use to cope, and the potential duration of surface activity, will be specific to the species and the desert where it lives. Whatever the thermal threat that occurs when an animal is on the surface, the animals of shifting-sand deserts have safety at hand; they can bury in the sand at their feet. Even those animals that have the most competent facultative thermoregulation have to abandon the surface if the thermal threat becomes unmanageable; there are no beetles or lizards in shifting-sand deserts that never bury.

A formal assessment of homeostatic priorities has been rare. In 2019, Pirtle and colleagues wrote that they knew ‘of only one study that has attempted experimentally to quantify the trade-off between thermoregulation and hydroregulation in a reptile species’ (p. 347), and that species was not a reptile of shifting-sand deserts (Pirtle *et al.*, 2019). Weaver and colleagues later showed that skin resistance to evaporative water loss, over a 3-mm diameter skin segment of a lizard, increased in a desiccating environment, compatible with saving body water, but again it was not a desert lizard (Weaver *et al.*, 2023). If we are to conserve the animals of shifting-sand deserts, we need much better knowledge of how they rank the homeostatic processes that are essential for their physiological welfare in those deserts. We suspect that they probably rank highest those processes that are required for reproduction.

## If desertification means more desert, does it aid the conservation of the animals of shifting-sand deserts?

Desertification attracts much more attention, from the public and the research community, than does the conservation of the animals in deserts. The reason is compelling: desertification affects human livelihoods (Ward, 2016). Desertification gives deserts a bad name. As Hadley and Szarek (1981, p. 747) put it, ‘the creation of new deserts or desert-like conditions (termed desertification) seriously reduces the land’s productivity and, in some cases, completely destroys it’. But does desertification affect the future of the animals of shifting-sand deserts? A superficial conclusion would be that those animals will not face conservation issues because, with progressive desertification, their habitat is growing. As with many issues in conservation biology, proper conclusions require more nuance, and concerns about desert animals cannot be dismissed. A fundamental problem is that the desertification of land that was not previously a shifting-sand desert does not create the substrate that is required by sand-swimming animals; that substrate can be generated only by wind, and many years of it. Also, the responses of plants to rainfall events in areas that have become desertified are not the same as the responses of the plants of shifting-sand deserts (Seely, 1991). Justifiable concern about desertification does not absolve us of the responsibility to conserve natural deserts, including their animals. Further, we shall argue that the conservation of the animals of shifting-sand deserts, traditionally the domain of ecology, requires a proper understanding of the physiology of those animals (Mitchell *et al.*, 2026).

In the next sections, we summarise some of the lessons that we have learnt about the animals of shifting-sand deserts, and possible approaches to their conservation.

### Desertification seems irresistible

Over geological time, the boundaries of the shifting-sand deserts of the world have expanded and contracted, primarily in response to cyclic natural changes in climate (Ward, 2016). Just 7000 years ago, the Sahara was a vegetated savannah, even if it was an arid savanna (Jürgens *et al.*, 2025). In the Holocene, and particularly in the last 150 years, anthropogenic changes have been imposed on those natural changes. Almost entirely, those anthropogenic changes have been unidirectional; they have created new desert-like conditions. Unlike the cyclic natural changes, the anthropogenic changes may be irreversible. According to Le Houérou (1996, pp. 168–169), ‘desertification continues to progress at a steady pace while anti-desertification programmes have achieved little’. However, the desertification that is happening now does not mimic, nor even approximate, the creation of a shifting-sand desert. Our aeolian shifting-sand deserts have taken tens of thousands (Twidale *et al.,* 2001) to tens of millions (Ward *et al.*, 1983) of years to form. Desertification increases desert quantity but does not maintain desert quality. Most perversely, some attempts to combat desertification could compromise the animals of shifting-sand deserts.

The underlying causes of recent desertification have been the expansion of the human population and the development that is required to house/feed/transport the population. That development has resulted in changes in the atmosphere and the natural environment. It would be convenient if we could attribute desertification in a straightforward way to climate change, or to overgrazing by domestic livestock, or to overcultivation. The catastrophic desertification of the area around the Aral Sea, and the consequent extension of the Karakum Desert in central Asia, undoubtedly was caused primarily by excess withdrawal of water, for cultivation purposes, from the two rivers that feed the Sea (Ward, 2016). Climate change and agricultural practices have contributed to desertification, but their consequences have been complex and locally variable. Direct analysis is complicated by the consequences of droughts, which are a natural, but temporary, occurrence in desert environments (Le Houérou, 1996). To dissect out the likely effects of climate change, long-term studies in which the effects of land abuse are absent, or can be eliminated statistically, would be required. No one has done such studies prospectively, but some natural experiments can be informative, even if they are anecdotal. What now is the Tsau/Khaeb National Park of Namibia was a forbidden area (‘sperrgebiet’), from which all human activities were excluded. That exclusion was not a conservation measure, but was to enhance security around diamond mines on the coast. In the period since 1908, and despite temporary emergency access to cattle in the drought years during the 1960s to early 1980s, there has been no measurable degradation of the dominant desert shrubs in the area (Jürgens *et al.*, 2025). Thus, climate change has not yet caused desertification there. By contrast, and despite more moisture and lower winds, in adjacent unprotected areas, those shrubs have been replaced by bare sand with occasional tussocks of desert grass that are resistant to wind-driven sandblasting. The desertification of those unprotected areas likely has been the consequence of mining activity and overgrazing by domestic livestock (Jürgens *et al.*, 2025). Mining typically causes severe but localised landscape degradation, but the degradation from mining exploration can be much more extensive. Exploration of the southern Simpson Desert in Australia left behind 880 visible crossings of sand dunes and 800 km of tracks (Graetz & Pech, 1987).

Although overgrazing by any large domestic animal species can lead to desertification (Hadley & Szarek, 1981; Ward, 2016), camel overgrazing is the most pernicious. Camels are well equipped physiologically to graze in arid environments that are sparsely vegetated. In the United Arab Emirates, for cultural reasons, camel production has been subsidised and camels have had free access to desert areas (El-Keblawy *et al.*, 2009). When exclosures that excluded camels but not native antelope were erected in those desert areas, vegetation cover and especially that of palatable species, increased substantially over 7 years (El-Keblawy *et al.*, 2009). In another desert with some vegetation, even in the absence of camel grazing, reptile diversity and abundance was lower on grazed farmland than on adjacent protected areas (Wasiolka & Blaum, 2011). In the Simpson Desert, the sinking of boreholes in the dune fields in the 1980s made intensive cattle grazing possible. After 20 years, the expected degradation of the desert vegetation had not occurred, perhaps because the grazing was managed to prevent overgrazing (Fensham *et al.*, 2010). In Namibia, the desertification of grasslands has occurred, but Ward and Ngairorue (2000) concluded that grazing has not been the cause.

When areas that have suffered anthropogenic degradation become protected, the fauna can recover faster than do the flora (Bartholomew & El Moghrabi, 2018). In the Desert Tortoise Research Natural Area in the Mojave Desert, USA, protected areas were created that excluded recreational off-road vehicles and sheep. Fifteen years later, the vegetation had not recovered fully, but desert lizards were much more abundant in the protected area than they were in adjacent areas, and the desert spiny lizard *Sceloporus magister* (primarily arboreal and not a shifting-sand species) was found only inside the protected area (Brooks, 1999).

Recently, there have been attempts to stop shifting-sand deserts from penetrating into adjacent areas by the planting of vegetated barriers (‘green walls’ or ‘shelterbelts’) in the desert sand. Those attempts were inspired by the extraordinary effectiveness of some natural barriers to the movement of the dunes of shifting-sand deserts. For example, the ephemeral Kuiseb River, though it may not flow at all in some years, has completely arrested the northwards migration of the central Namib dune sea (Eckardt *et al.*, 2013). Anthropogenic barriers are intended both to bind the sand and to reduce the winds that transport sand close to the surface. Many have had limited success, or no success. Eighty percent of the trees planted in the ‘Great Green Wall’ of north Africa died within 2 years (Zhang *et al.*, 2023). The planting of African buffel grass *Cenchrus ciliaris* to stabilise the mobile sands of the Sonoran Desert (USA) has been a disaster, particularly because it has acted as a tinder for fires that the native vegetation, including the iconic saguaro *Carnegiea gigantea*, cannot withstand (Marshall *et al.*, 2012). The use of bulldozers to counteract the fires will compound the problem for animals living in the top layers of the desert sand (Moroney & Rundel, 2013).

The green walls or shelterbelts in China seem to have been more successful (Ward, 2016), though it is too early to judge the success of China’s own Great Green Wall, around the Taklamakan Desert (Tan & Li, 2015). Vegetation along the margins of the Taklamakan Desert already have converted it to a net carbon sink, at least in the wet season (Noor *et al.*, 2026). Perhaps the oldest of those green walls in China was created to protect the Baotou–Lanzhou railway against windblown sand from the Tengger Desert. That barrier reduced the near-surface wind speed by 54% and, after 46 years, had set up a new animal habitat with 28 bird species and 50 insect species in residence that were not there previously (Li *et al.*, 2004). Ominously, though, China’s programme of grassland restoration and afforestation has led to a reduction in water availability in its northwestern arid zone (An *et al.*, 2025). In 2010, Wang *et al.* (2010, p. 13) concluded that ‘although the large-scale afforestation program may have had some beneficial effects on reducing dust storms and controlling desertification in China, the results of our analysis suggest that the importance of this project seems to have been overstated’. A more recent analysis concluded that shelterbelts generally had increased ecosystem services, but had been least effective in the drier parts of the Chinese deserts (Guan *et al.*, 2026).

While there are several reports of the consequence of those barriers for the areas that they are meant to protect from desertification, we know of no reports on the consequence of those barriers for the animals of the shifting-sand deserts that are behind the barriers. In some cases, a barrier would be to the detriment of the existing desert. For example, the establishment of a barrier to stop the eastward migration of the Namib Desert would likely lead to the demise of the entire animal biota of that desert, because the ecosystem depends on detritus that is blown into the desert from highlands that are hundreds of kilometres to its east (see Mitchell *et al.,* 2026).

### Shifting-sand deserts are irreplaceable

It is a disappointment for the industry, but fortunate for the shifting-sand deserts themselves, that desert sand is unsuitable for construction. Fifty billion tonnes of aggregate, much of it sand, goes into construction each year (Beiser, 2019). Despite housing much of the huge Arabian Desert, Saudi Arabia has to import sand for construction purposes (Zhong *et al.*, 2022). The reason that desert sand is unsuitable for construction is that it is too spherical, which reduces the surface irregularities that are necessary to lock sufficiently in concrete. The granules are spherical because they have been blown and tumbled in wind, sometimes for tens of millions of years. Especially in places where wind direction and strength change over time, the grains of dune sand are not uniform in size. Grain diameter tends to be bimodally distributed, with assemblies of coarse grains overlying the finer grains (Tsoar, 1990). Sand structure varies with height over a dune, and the sand is compacted much more on the windward side of a dune than it is on the leeward side. Thus, the reconstruction of an aeolian sand dune system after it has been damaged by human activity is a formidable task. So formidable, according to Ryberg and Fitzgerald (2015, p. 120), that it may be impossible: it ‘would require multi-scale landform restoration from the hundreds of hectares of interconnected dunefields down to the composition of sand grain size at local sites’. The conservation goal has to be to prevent damage, rather than to remediate damage.

To the mining activities and the grazing (and trampling) by large domestic herbivores that can damage the shifting dunes, we should add industrial roads, railways, powerlines and pipelines (Lovich & Bainbridge, 1999), as well recreational use of off-highway vehicles (OHV) on dunes, especially in the deserts of California, USA (Robinson & Barrows, 2013). As a result of OHV and other disturbances in those Californian deserts, the future is bleak for at least six species of the *Uma* genus of quiescent burying lizards (Robinson & Barrows, 2013). It also is bleak for endemic Californian sand-swimming beetle species with limited habitat ranges, like the *Ciervo aegialian* scarab beetle *Aegialia concinna* (Gordon & Cartwright, 1988) and the San Joaquin dune tenebrionid beetle *Coelus gracilis* (Doyen, 1976), confined to the Monvero Dunes. The damage that vehicles can cause on sand dunes is obvious. Even when vegetation is not damaged, the traverse of a vehicle can disrupt the terrain that might be occupied by surface-active animals or quiescent burying animals, and compaction of the sand will make burying difficult for sand-swimming animals. The temperature profile below the sand surface (Fig. 8), and the penetration of water and water vapour, will also be disturbed (Liddle & Moore, 1974). The noise from OHVs has caused the unnatural emergence of buried spadefoot toads (Brattstrom & Bondello, 1983; Lovich & Bainbridge, 1999) and OHV activity at night is likely to disrupt the seismic location of prey by the Namib golden mole. The noise of dune buggies was sufficient to cause neurophysiological hearing loss in the Mojave fringe-toed lizard *U. scoparia*, potentially affecting its ability to both locate prey and avoid predators (Brattstrom & Bondello, 1983). Lovich and Bainbridge (1999) considered the impact of OHVs in the Californian deserts to be more severe than that of grazing or mining. The conservation of the animals of shifting-sand deserts will require better regulation of OHV activity and the establishment of exclusion zones.

More extensive than any impact of OHVs is an activity that is regarded by many as heroic: the ‘greening’ of the deserts. The animals studied by Cowles (1941) were unearthed in desert areas that were being excavated for conversion to agriculture. Especially in countries that have deserts and have exhausted other land that is available for agriculture, the deserts present an opportunity ‘to derive revenue from sand normally regarded as unproductive wasteland and easily eroded soil’ (Tsoar, 1990, p. 148). Shifting-sand deserts are well suited to drip irrigation, especially for orchards, and many productive orchards have been established in what was shifting-sand desert. The aim always has been to ‘derive revenue’, not to combat starvation. The greened deserts tend not to be used to produce staple foods. Many more are planned, particularly in Saudi Arabia (Ghanem & Alamri, 2023; https://www.unthinkablebuild.com/saudi-arabia-is-building-the-longest-river-in-the-desert/). We do not regard the greening as ‘reclamation’ of the desert but as exploitation of the desert, in principle no different to the clearing of South American rain forest for cattle farming or of the Malaysian forest for the production of palm oil. The forests are more likely to recover in the unlikely event that their exploitation was stopped. We cannot envisage that shifting-sand deserts will recover from such exploitation; they are irreplaceable.

An indirect anthropogenic threat to shifting-sand deserts comes from another form of ‘greening’, namely that resulting from greenhouse gases. The shifting-sand deserts are vast, and large regions of the world’s deserts still show little negative impact of human abuse (Ward, 2016). But even if direct human disturbance to the desert itself does not occur, the shifting-sand deserts of the future are unlikely to be like those of the past. The primary cause will be the change in climate (Ward, 2016). That process is exemplified by the greening of the Thar Desert in India and Pakistan. Climate change has resulted in a westward expansion of the monsoon belt into the desert, creating unprecedented rainfall and recharge of aquifers (Mishra *et al.*, 2025). In the Thar Desert during the 21st Century, there has been a 38% increase in normalised difference vegetation index (NDVI), and a 74% increase in crop area (Mishra *et al.*, 2025). Invasive alien plants contribute to the NDVI, and there has been a plague of feral dogs (Misher *et al.*, 2025). The greening has been accompanied by urbanisation; 10 million people have moved into the Desert (Mishra *et al.*, 2025). We are unaware of any reports of what has happened to the animals that formerly occupied the desert. The Negev Desert has also had a sustained increase in precipitation in the 21st century but with fewer consequences for human habitability (Mishra *et al.*, 2025).

Any prediction on the future of shifting-sand deserts under climate change is complicated by anomalous and competing processes. Between 1980 and 2018, the global land area in which multiyear (>5 y) droughts occurred increased by an average of 50 000 km^2^·y^−1^ (equivalent to an increase in area by the size of California every 10 years), but multiyear droughts did not occur in any of the world’s major deserts (Chen *et al.*, 2025). From 1948 to 2017, woody vegetation in the Namib Desert (almost entirely in the beds of ephemeral rivers) increased, rather than declined under global warming and drying (Rohde *et al.*, 2019). The enhancement of photosynthesis due to an increase in the atmospheric concentration of carbon dioxide (the CO_2_ fertilisation effect) already has resulted in the greening of vegetation in excess of that anticipated from rainfall changes (Donohue *et al.*, 2013). The greening effect will accelerate as the carbon dioxide concentration continues to rise, and is expected to be evident particularly in desert vegetation (Donohue *et al.*, 2013). But by the end of the century, while global precipitation is expected to increase (Konapala *et al.*, 2020), the Palmer drought severity index, which assesses water available to plants, predicts severe to extreme drought for the shifting-sand deserts of southern Africa, North America, the Middle East, Australia and the Thar Desert (Dai, 2012). Anomalously, the deserts of central China, and, except for its western fringe, the Sahara Desert, escape that fate. Although it is a lack of precipitation that defines deserts, the vegetation of shifting-sand deserts is still driven by rain because the plants require above-average rainfall events (‘big rains’) to germinate (Smith & Morton, 1990; Southgate *et al.*, 1996). Those events occur only sporadically, but if they fail, the vegetation of shifting-sand deserts, and the welfare of the animals that depend on the plants, inevitably must decline. The shifting-sand deserts that we know today not only are irreplaceable in their geomorphology but those that survive are unlikely to be the deserts that we know.

### A fortress in the desert sand

We have related (Section A home in the sand) how many animals have made a home in shifting-sand deserts. A better analogy might be that they have found a fortress in the sand. It is a home that is unlikely to be invaded by other animals, and it is a home that many animals will find difficult to leave with benefit. Those animals have adapted and become habitat specialists (Greenville & Dickman, 2009).

We have discussed how special animals, or groups of animals, can move in and on the loose sand of shifting-sand deserts, and how they can avoid death from heat and cold. The animals range taxonomically from invertebrate nematodes to vertebrate marsupial moles. Their physiology is admirably suited to their environment; the desert sand is not hostile to them. How they arrived at this favourable state still is a matter of debate. Has each species evolved a genetic make-up that equips it for life in a shifting-sand desert? Is there a set of shifting-sand desert genotypes? If so, conservationists will be relieved of the task of excluding alien animals from shifting-sand deserts because those animals will not have a desert genotype. Or has the sequence of events been phenotypic? Were the animals then pre-adapted and so pre-equipped with physiological competencies that allowed them to cope with the shifting-sand environment (Henschel *et al.*, 2001), or have the adjustments occurred entirely within the shifting-sand deserts? If animals can be pre-equipped to enter shifting-sand deserts, why is there little evidence for new immigrants? Even animals that live close to shifting-sand deserts might not have the pre-adaptations that are necessary to enter those deserts. For example, the iguanid lizard *Sceloporus occidentalis* is resident in the Santa Ana mountains just west of the shifting-sand deserts of California. That species cannot survive without free water (Munsey, 1972) and so, we contend, could not establish itself in the dry desert.

S.D. Bradshaw, an Australian expert on desert reptiles, has argued forcibly against the idea that there is a desert genotype in reptiles (Bradshaw, 1988). He has been ‘unable to discern any unique features which, at one and the same time, distinguish desert reptiles from close relatives restricted to mesic and humid environments, and also account for their manifest ability to survive and reproduce in environments which are amongst the most extreme found on earth’ (Bradshaw, 1988, p. 170). Rather, he argues, there is a repertoire of physiological competencies, autonomic and behavioural, that is available to all reptiles, and that repertoire equips them to adjust to their environment. Desert reptiles have selected those adjustments that are appropriate to the desert environment and then used them successfully. That process conforms to pre-adaptation. In the case of desert mammals, others have proposed a strong adaptive genetic basis to the occupation of deserts (Rocha *et al.*, 2021). Australian marsupial moles and Namib golden moles (see Fig. 2) have co-evolved remarkably similar morphology, including sleek pelage, an absence of external appendages, and shovel-like front limbs. They are phylogenetically so distant, however, that we cannot accept that their convergent evolution relies on a shared set of genes.

What happens to animals that are adapted to a particular environment if the environment changes in such a way that they no longer can function successfully in it? That will be an inevitable consequence of climate change for some animal communities. One option in other biomes is that the animals migrate to a more acceptable environment. That migration may be to higher altitude or latitude. The altitude variation in shifting-sand deserts is confined to the height of a sand dune, at most a few hundred metres, so will be of little help to animals to gain altitude. Also, except for the Californian deserts, the shifting-sand deserts of the world are not contiguous, so terrestrial animals cannot move from one shifting-sand desert to another. An example from the Namib Desert reveals that movement even within one desert may be difficult. Within the Namib Desert, the northern and central sand seas appear very similar but are separated by gravel plains. The sand dune lizards *G. skoogi* and *M. anchietae* co-occur in the northern sand sea, but *G. skoogi* is absent from the central sea (Mitchell *et al.*, 1987). The species appears to have been unable to cross a few hundred kilometres of firm substrate on the gravel plains. The sand sea fortress might be as effective at keeping its residents in as it is at keeping aliens out.

If migration is not an option, can anything be done to conserve the animals of a shifting-sand desert in the face of a change to their environment? They may not have the pre-adaptations that are necessary to cope with a worsened environment (Seely, 1979; Nagy, 1988, Smith & Morton, 1990).There will be no conservation panacea (Zhang *et al.*, 2023), not least because there is no single physiological attribute, or even group of attributes, that seems to equip animals to live in shifting-sand deserts (Stevenson, 1985; Nagy, 1988). According to Louw (1971, p. 123), ‘it is seldom a single dramatic factor which allows animals to survive in the desert but rather a series of small adaptations which, acting in concert, ensure perfect dovetailing of the animal with its environment’. Indeed, Nagy (1988, p. 209) later concluded ‘that a rich diversity of desert adaptations remains to be discovered’. If there is a single dramatic factor that has enabled mammals throughout the world to have survived over the ages in shifting-sand deserts, that factor is an ability to access the subsurface environment (Benevento, 2025; Pinkert *et al.*, 2025), at which the animals of shifting-sand deserts are expert. Nevertheless, the conservation of the animals of shifting-sand deserts will have to be conducted niche-specifically (Dawson *et al.*, 1989), that is, case by case, and the data that would inform decisions may not have been gathered yet. That constraint will be amplified as the animals of shifting-sand deserts face the effects of climate change, because the effects of climate change on macroclimates and microclimates might be quite different (Pirtle *et al.*, 2019). Conservation of one species, however, sometimes leads to secondary conservation of other species that are ‘under its umbrella’ (Ward, 2016).

## Supplementary Material

Web_Material_coag057

## Data Availability

No data were generated or analysed during this work.
