# Supplementary material for: Physiology and the future of animals in shifting-sand deserts: living, moving and thermoregulating in the sand
Source: Conserv Physiol. 2026 Aug 20;14(1):coag057. doi: 10.1093/conphys/coag057 (PMC13491554; doi:10.1093/conphys/coag057)
Supplement: Supplementary file 1 [file web_material_coag057.zip › Mitchell_et_al._Part_1_supplementary_material_second_revision.docx]

**Part 1 supplementary material**

Appendix 1: The biomechanics of sand-swimming and quiescent burial

Of the 25 families of lizards, ten contain species that have absent or reduced limbs, and almost all of those species occupy subsurface habitats (Benesch and Withers, 2002), although the occupation of subsurface habitats is not always associated with limb reduction in squamates (Camaiti *et al.,* 2021). When a lizard that has legs swims in the sand, it first dives or plunges into the sand. The morphology of its snout often facilitates the entry of the animal into the sand (Mosauer, 1932; Louw, 1971; Arnold, 1995; Etheridge, 2000; Edwards *et al.,* 2016; Evans *et al.,* 2016; Bergmann and Berry, 2021) The lizards use quadrupedal movement when they are on the surface, and to enter the sand they use force generated by the legs, especially the hindlegs. At least for the toad-headed agama *Phrynocephalus mystaceus*, which inhabits sandy deserts of central Asia, toe fringes are an impediment to sand entry (Zheng *et al.,* 2024). But the lizards abandon the use of both forelegs and hindlegs once they are buried (Arnold, 1995). Thus lizards and sand-swimming snakes, like *Sonora occipitalis*, have similar movements in the sand (Mosauer, 1932). X-ray imaging showed that all four legs of the sandfish were adducted completely when it swam in the sand (Maladen *et al.,* 2009) or in glass particles that simulated its sand medium (Maladen *et al.,* 2011). So too were the limbs of the Australian skink *Lerista elegans* (Benesch and Withers, 2002). Though it stays close enough to the surface to qualify for quiescent burial rather than sand-swimming, the ocellated skink *Chalcides ocellatus* uses undulating movement with adducted legs to progress through the sand (Catena and Hembree, 2014). Using fast NMR imaging of sandfish below the sand, Baumgartner and colleagues (2008) observed that the limbs were not adducted, but Maladen *et al.,* (2009) consider those observations to be artefacts.

Legless lizards, like *Acontias* of the Kalahari Desert (Huey *et al.,* 1974), and snakes, use an undulating movement to wriggle into the superficial layers of the sand, where they remain concealed but within a few body diameters of the surface, and do not move more than a few body lengths from the burial site; they exhibit the “quiescent burial” of Pough (1970). On the surface, the legless Zarudny's worm lizard *Diplometopon zarudnyi,* of Middle Eastern deserts, curls its tail and anchors the tip in the sand, to assist with forward locomotion; to bury, it plunges its shovel-shaped snout and then wriggles into the sand (Winser, 1989). The sidewinder rattlesnake *Crotalus cerastes* does not have a snout that is suitable to plunge into the sand; it buries its tail before it buries its head, and then does not move (Mosauer, 1932). The 6-13 g shovel-nose snake *Chionactis occipitalis*, which spends most of its life buried, moves through sand with the same undulating movement as do lizards (Norris and Kavanau, 1966). Even though Sharpe and colleagues (2015) considered the longer body and reduced surface friction of *Chionactis* to make it a more-efficient sand-swimmer than is the sandfish, the snake swims at one-tenth of the speed of sandfish (Astley *et al.,* 2020), which has been measured at an astounding 300 mm.s^-1^ in the laboratory (Baumgartner *et al.,* 2008). Quiescent burial also is characteristic of those species of fringe-toed lizards in the genus *Uma* of the deserts of north-west America that live in loose sand, although some *Uma* lizards burrow in firmer sand (Pough *et al.,* 1978). The Coachella fringe-toed lizard *U. inornata* (Figure 2), a resident of deep wind-blown sand (Barrows, 1997) with a snout-to-vent length of 70 – 120 mm, buries just 5 - 40 mm below the sand surface (Pough, 1970).

Unlike the true sand-swimming lizards, lizards that engage in quiescent burial, except the ocellated skink *Chalcides ocellatus*, continue to use their legs to propel themselves over the short distances that they move in the sand (e.g. *Lerista lineopunctulata,* Benesch and Withers, 2002; *L. labialis,* Greenville and Dickman, *2009* and perhaps all *Lerista* species, Morinaga and Bergmann, 2020), though it may be only their forelegs (Pough, 1970). Mojave fringe-toed lizards *U. scoparia* use their hind legs, and the way that they coordinate the movement of the trunk, tail, and hindlegs during quiescent burial has been studied electromyographically (Jayne and Daggy, 2000). The total burial time decreased markedly with ambient temperature, but the mean was less than 5 s at ambient temperatures between 23°C and 39°C (Jayne and Daggy, 2000).

Namib golden moles use a cyclic movement to swim in the sand, but it is not the lateral undulation that is used by sand-swimming reptiles. Burying starts with a vertical plunge of the head, followed by digging with the forelegs, and then the propulsion of a bent body by the hindlegs. Cinefluorographical experiments in the laboratory, with the moles in semolina (Gasc *et al.,* 1986), showed that once the mole is buried, it begins its cycle with a short buttress phase, supported on all four limbs with the back dorsiflexed, which loosens the surrounding substrate. The mole then reaches forward with both of its powerful forelimbs, excavates substrate from under the head and pushes the sand under the abdomen. Then, using a synchronized extension of forelimbs and hindlimbs, the mole makes a double thrust forward, and the substrate moves first over the head and back and then under the chest. The hindlimbs then anchor the mole for the next cycle.

References

Arnold EN (1995) Identifying the effects of history on adaptation: origins of different sand‐diving techniques in lizards. *J Zool.* 235:351-388.

Astley HC, Mendelson III JR, Dai J, Gong C, Chong B, Rieser JM, Schiebel PE, Sharpe SS, Hatton RL, Choset H *et al.,* (2020) Surprising simplicities and syntheses in limbless self-propulsion in sand. *J Exp Biol.* 223:jeb103564.

Barrows CW (1997) Habitat relationships of the Coachella Valley fringe-toed lizard (*Uma inornata*). *Southwest Nat.* 42:218-223.

Baumgartner W, Fidler F, Weth A, Habbecke M, Jakob P, Butenweg C, Böhme W (2008) Investigating the locomotion of the sandfish in desert sand using NMR-imaging. *PLOS One* 3:e3309.

Benesch AR, Withers PC (2002) Burrowing performance and the role of limb reduction in *Lerista* (Scincidae, Lacertilia). *Senckenbergiana lethaea*. 82:107-114.

Bergmann PJ, Berry DS (2021) How head shape and substrate particle size affect fossorial locomotion in lizards. *J Exp Biol.* 224:jeb242244.

Camaiti M, Evans AR, Hipsley CA, Chapple DG (2021) A farewell to arms and legs: a review of limb reduction in squamates. *Biol Rev.*96:1035-1050.

Catena AM, Hembree DI (2014) Swimming through the substrate: the neoichnology of *Chalcides ocellatus* and biogenic structures of sand-swimming vertebrates. *Palaeontol Electron.*17:17.3.37A.

Edwards S, Herrel A, Vanhooydonck B, Measey GJ, Tolley KA (2016) Diving in head first: trade-offs between phenotypic traits and sand-diving predator escape strategy in *Meroles* desert lizards. *Biol J Linn Soc.*119:919-931.

Etheridge R (2000) A review of lizards of the *Liolaemus wiegmannii* group (Squamata, Iguania, Tropiduridae), and a history of morphological change in the sand-dwelling species. *Herpetol Monogr.* 14:293-352.

Evans JS, Eifler DA, Eifler MA (2017) Sand-diving as an escape tactic in the lizard *Meroles anchietae*. *J Arid Environ.* 140:1-5.

Gasc JP, Jouffroy FK, Renous S, Blottnitz FV (1986) Morphofunctional study of the digging system of the Namib Desert golden mole (*Eremitalpa granti* *namibensis*): cinefluorographical and anatomical analysis. *J Zool.* 208:9-35.

Greenville AC, Dickman CR (2009) Factors affecting habitat selection in a specialist fossorial skink. *Biol J Linn Soc.* 97:531-544.

Huey RB, Pianka ER, Egan ME, Coons LW (1974) Ecological shifts in sympatry: Kalahari fossorial lizards (*Typhlosaurus*). *Ecology* 55:304-316.

Jayne BC, Daggy MW (2000) The effects of temperature on the burial performance and axial motor pattern of the sand-swimming of the Mojave fringe-toed lizard *Uma scoparia*. *J Exp Biol.* 203:1241-1252.

Louw GN (1971) Water economy of certain Namib Desert animals. *S Afr J Sci.* 67:119 - 123.

Maladen RD, Ding Y, Li C, Goldman DI (2009) Undulatory swimming in sand: subsurface locomotion of the sandfish lizard. *Science* 325:314-318.

Maladen RD, Ding Y, Umbanhowar PB, Kamor A, Goldman DI (2011) Mechanical models of sandfish locomotion reveal principles of high performance subsurface sand-swimming. *J R Soc Interface* 8:1332-1345.

Morinaga G, Bergmann PJ (2020) Evolution of fossorial locomotion in the transition from tetrapod to snake-like in lizards. *Proc Roy Soc London Ser B Biol Sci.* 287:20200192.

Mosauer W (1932) Adaptive convergence in the sand reptiles of the Sahara and of California: a study in structure and behavior. *Copeia* 1932:72-78.

Norris KS, Kavanau JL (1966) The burrowing of the western shovel-nosed snake, *Chionactis occipitalis* Hallowell, and the undersand environment. *Copeia* 1966:650-664.

Pough FH (1970) The burrowing ecology of the sand lizard, *Uma notata*. *Copeia* 1970:145-157.

Pough FH, Morafka DJ, Hillman PE (1978) The ecology and burrowing behavior of the Chihuahuan fringe-footed lizard, *Uma exsul*. *Copeia* 1978:81-86.

Winser N (1989) The Sea of Sands and Mists. Century Hutchinson, London.

Zheng P, Liang T, Shi L (2024) Are toe fringes important for lizard burying in highly mobile sand? *Front. Zool.* 21:25.

....................................................................................................................................

Appendix 2: The colour of tenebrionid elytra

Colour plays no role in the direct radiant heat that is exchanged between the desert sand itself and an animal on it, because that radiation is in the infrared waveband and all animals are near-perfect absorbers in the infrared (Mitchell *et al.,* 2018). More than half of the energy in the solar spectrum also is from radiation in the infrared waveband (Stuart-Fox *et al.,* 2017). The colour of an animal affects only radiant heat transfer that is in the visible waveband.

That most tenebrionid beetles of the Namib Desert have black elytra while a few species, of similar mass, have white elytra allows us to assess the thermal consequences of being black in the desert sun. Edney (1971) showed that a beetle with a black elytron (*Onymacris rugatipennis*) heated faster than did one with a white elytron (*O. bicolor, then O.brincki* , Penrith,1975). Others (e.g. Bolwig, 1957; Hadley, 1970) also reported higher temperatures below black elytra than below white elytra, but the difference was smaller than the 10°C reported by Edney (1971), especially if the beetles were close to the sand surface (Turner and Lombard, 1990). Edney himself (1971) did not consider that the white colour provided a selectively important thermal benefit; he considered the white colour to be aposematic.

Though it may not be the only factor that affects the radiant heat transfer of beetles that have elytra of different colours (Turner and Lombard, 1990), the reflectivity of the cuticle in the visible waveband is likely to play a role. Henwood (1975) estimated that the reflectivity of the lighter parts of beetle cuticle was 50-60%, while the black parts reflected only 23%. The wax blooms that are extruded on to the carapace by some tenebrionid beetle species, which reduce water loss (see Mitchell *et al.,* 2026), and which have a range of colours including white (McClain *et al.,* 1985), reflected only 11 to 26% of the visible waveband, but that may be enough to extend the period of diurnal activity (McClain *et al.,* 1991). The silvery appearance of Saharan silver ants *Cataglyphis bombycina* results from an array of triangular hairs that have extraordinary optical properties. The array increases reflectivity in the visible and near infrared waveband, and so reduces the impact of direct solar radiation, while also increasing the emissivity at longer infrared wavelengths, which enhances radiant loss from the ants to their surroundings (Shi *et al.,* 2015).

References

Bolwig N (1957) Experiments on the regulation of the body temperature of certain tenebrionid beetles. *J Entomol Soc S Afr*. 20:454-458.

Edney EB (1971) The body temperature of tenebrionid beetles in the Namib Desert of southern Africa. *J Exp Biol.* 55:253-272.

Hadley NF (1970) Micrometeorology and energy exchange in two desert arthropods. *Ecology* 51:434-444.

Henwood K (1975) A field‐tested thermoregulation model for two diurnal Namib Desert tenebrionid beetles. *Ecology* 56:1329-1342.

McClain E, Seely MK, Hadley NF, Gray V (1985) Wax blooms in tenebrionid beetles of the Namib Desert: correlations with environment. *Ecology* 66:112-118.

McClain E, Kok CJ, Monard LAG (1991) Reflective wax blooms on black Namib desert beetles enhance day activity. *Naturwissenschaften* 78:40-42.

Mitchell D, Snelling EP, Hetem RS, Maloney SK, Strauss WM, Fuller A (2018) Revisiting concepts of thermal physiology: predicting responses of mammals to climate change. *J Anim Ecol.* 87:956-973.

Mitchell D, Seely MK, Maloney SK (2026) Physiology and the future of animals in shifting‑sand deserts: Respiration, energetics, and water balance. *Conserv Physiol.*, in press.

Penrith ML (1975) The species of *Onymacris* Allard (Coleoptera: Tenebrionidae). *Cimbebasia* 4:47-97.

Shi NN, Tsai CC, Camino F, Bernard GD, Yu N, Wehner R (2015) Keeping cool: Enhanced optical reflection and radiative heat dissipation in Saharan silver ants. *Science* 349:298-301.

Stuart-Fox D, Newton E, Clusella-Trullas S (2017) Thermal consequences of colour and near-infrared reflectance. *Philos Trans R Soc B Biol Sci.* 372:20160345.

Turner JS, Lombard AT (1990) Body color and body temperature in white and black Namib desert beetles. *J Arid Environ*.19:303-315.

............................................................................................................................
